# Supplementary material for: Detecting macroevolutionary genotype–phenotype associations using error-corrected rates of protein convergence
Source: Nat Ecol Evol. 2023 Jan 5;7(1):155–70. doi: 10.1038/s41559-022-01932-7 (PMC9834058; doi:10.1038/s41559-022-01932-7)
Supplement: Supplementary file 1 — Supplementary Methods, Texts 1–17, Figs. S1–S16 and References. [file 41559_2022_1932_MOESM1_ESM.pdf]

nature ecology & evolution

Article https://doi.org/10.1038/s41559-022-01932-7

Detecting macroevolutionary genotype–phenotype associations using error-corrected rates of protein convergence

In the format provided by the authors and unedited

Supplementary Information for

Detecting macroevolutionary genotype-phenotype associations using error-corrected rates of protein convergence

Kenji Fukushima and David D. Pollock

List of Supplementary Materials:

Supplementary Methods
Supplementary Texts 1–17
Supplementary Tables S1–S10 (separate file)
Supplementary Figs. S1–S16
Supplementary References
Supplementary Dataset (separate file on Dryad: https://doi.org/10.5061/dryad.tx95x6b0v)[94]

Supplementary Methods

Animal gene sets. A dataset of amalgamated cross-species transcriptomes[44] was generated for 21 vertebrate genomes in Ensembl 91[98] (Supplementary Table 10). To ensure compatibility, the same versions of protein-coding sequences were also used for the convergence analysis. Completeness of genome assembly was evaluated using BUSCO v4.0.5[99] with the single-copy gene set of 'tetrapoda_odb10' (Supplementary Table 10). A species phylogenetic tree previously downloaded from TimeTree[95] was used[44]. Orthogroups were classified by OrthoFinder v2.4.1[32,100]. Orthogroups containing more than three genes were analyzed further. During the analysis of this dataset, a protein size–dependent change in measured convergence rates was observed (Supplementary Fig. 16) but was determined to be an artifact; ω_C was shown to be more robust to the bias than the other metrics (Supplementary Text 16).

Sequence retrieval from public databases. Gene sets for previously confirmed cases of molecular convergence and horizontal gene transfer events (HGTs) were generated based on previous reports with increased taxon sampling (Supplementary Table 3 and Supplementary Text 17). With GenBank accession numbers for ATPalpha1, Prestin, PEPC, and PCK homologs (Supplementary Dataset[94]), coding sequences (CDSs) were retrieved using the 'accession2fasta' function of CDSKIT. Lysozyme sequences were downloaded as GenBank files from NCBI and were converted to fasta files with the 'parsegb' function of CDSKIT. For the retrieval of the mitochondrial genome, a custom python script was used to select balanced numbers and lineages of foreground and background species (Supplementary Dataset[94]). Orthogroup CDS files for og3737 (leucine-tRNA ligase), og9103 (pentatricopeptide repeat protein), and og9298 (pentatricopeptide repeat protein) for the HGT events in *Cuscuta* were obtained from a previous report[31], and genes leading to unrealistically long branches were excluded. HGTs in the other parasitic lineage Orobanchaceae were also analyzed in the same report, but HGTs in *Cuscuta* were used for performance evaluation because the donor lineage was unequivocal in several genes.

Sequence retrieval from plant gene sets. Gene sets were downloaded from public databases for the retrieval of CDSs encoding digestive enzyme homologs (Supplementary Table 10). Transcriptome assemblies were used as a part of gene sets. For *Drosera adelae*, *Nepenthes* cf. *alata*, and *Sarracenia purpurea*, previously assembled transcriptomes were used[27]. The transcriptome assembly of *Rhododendron delavayi* was generated from publicly available RNA-seq data (NCBI BioProject ID: PRJNA476831) with Trinity v2.8.5[101] after pre-processing with fastp v0.20.1[102] (Supplementary Dataset[94]). Subsequently, open reading frames (ORFs) were obtained with TransDecoder v5.5.0 (https://github.com/TransDecoder/TransDecoder). The longest ORFs among isoforms were extracted with the 'aggregate' function of CDSKIT. The completeness of assembly was evaluated using BUSCO scores with the single-copy gene set of 'embryophyta_odb10' (Supplementary Table 10). Finally, digestive enzyme homologs were retrieved by TBLASTX v2.9.0 searches against all gene sets with an E-value cutoff of 0.01 and >50% query coverage[103].

Characterization of protein-coding sequences. Coding sequences were used for RPS-BLAST v2.9.0 searches[103] against Pfam-A families[104] (released on April 30, 2020) with an E-value cutoff of 0.01 to obtain protein domain architectures. The numbers of transmembrane domains were predicted by TMHMM v2.0[105]. The numbers of introns in protein-coding sequences were extracted from GFF files downloaded from Ensembl. Further gene annotations were obtained using Trinotate v3.2.1 (https://github.com/Trinotate/Trinotate.github.io/wiki).

Plant species tree. Orthogroup classification was performed with OrthoFinder v2.4.1[32]. Stop codons and ambiguous codons were masked as gaps using CDSKIT. In-frame multiple sequence alignments of single-copy orthologs were generated by MAFFT v7.455 with the --auto option[75] and tranalign in EMBOSS v6.6.0[106]. Ambiguous codon sites were then removed by ClipKIT v0.1.2 with the default parameters[107]. After the concatenation of trimmed sequences, a maximum-likelihood phylogenetic tree was reconstructed by IQ-

TREE v2.0.3 with the GTR+G nucleotide substitution model[80,108]. The tree was rooted using *Amborella trichocarpa* as an outgroup. The divergence time of the species tree was estimated using mcmctree in the PAML package v4.9[109]. The priors and parameters were chosen according to the mcmctree tutorial (http://abacus.gene.ucl.ac.uk/software/paml.html). Fossil calibrations were adopted from a previous study[110].

Analysis of gene duplications. Gene duplications on gene trees were inferred by a species-overlap method, as explained above. Branches following gene duplication events are annotated as D branches. Note that, if one copy after the gene duplication is not included in the dataset due to poor gene annotation or other reasons (see Supplementary Table 10 for gene completeness), the gene duplication node is lost and the branch that should have been classified as a D branch is combined with its parent branch. Such a bias would cause contamination from the D branch to the S branch, but the effect would be negligible because S branches are much more numerous than D branches and the opposite does not occur. Pairs of branches following two independently occurred gene duplications were extracted as DD branch pairs. In the genome-scale analysis, DD branch pairs may be connected to different species, or to the same species if successive duplications happen in the lineage. Nevertheless, paralogous gene lineages were compared in all cases. While the analysis of DD pairs was designed to characterize convergent gene duplications, one may wish to analyze the convergence of two copies generated by single gene duplication. In such an analysis, each copy must undergo speciation as soon as possible thereafter, since it is impossible to analyze convergence immediately after a duplication, where the branches are sisters to each other. Therefore, such an analysis will be best performed with a more densely taxon-sampled dataset to minimize the signal loss due to unanalyzable branches after duplications. Our methods are compatible with gene losses, but lost gene lineages lead to the absence of ancestor-descendant branches that could otherwise be analyzed and contribute to the informativeness of the data.

Data visualization. Phylogenetic trees were visualized using the python package ETE 3[87] and the R package ggtree[111]. General data visualization was performed with python packages matplotlib[112] and seaborn[113] as well as the R package ggplot2[114]. Boxplot elements of all figures are defined as follows: center line, median; box limits, upper and lower quartiles; whiskers, 1.5 × interquartile range.

Supplementary Texts

Supplementary Text 1. False positives in the detection of molecular convergence by topology-based methods. By taking advantage of the branch attraction potentially caused by molecular convergence, which may be detected as a form of site-specific likelihood supports for alternative tree topologies, Parker et al. reported that nearly 200 out of 2,326 orthologous proteins were convergently evolved between echolocating bats and whales[115]. However, thorough reexaminations of their methodology, which evaluates convergence by phylogenetic tree topology without reconstructing ancestral sequences and substitutions, revealed that most of the reported genomic signatures for molecular convergence were false positives that often lack convergent substitutions (98/117 genes listed as convergent between bats and dolphins), highlighting the need to directly evaluate convergent substitutions rather than indirect signatures such as site-specific likelihood supports[11,12].

Supplementary Text 2. Phylogenetic combinations of substitutions. When two separate lineages each experience a codon substitution at the same position in a protein, we call these paired substitutions (Supplementary Fig. 1c). Paired substitutions may be of interest regardless of the codons involved, particularly if there are coincident bursts of paired substitutions along two lineages and especially if the burst involves more nonsynonymous than synonymous changes. Furthermore, if nonsynonymous paired substitutions result in the same amino acid, they are considered convergent substitutions at the amino acid level, potentially of great interest if similar selective pressures have driven the convergent events. Here, we use the classic definition of convergent evolution, that is when two biological traits in two separate lineages independently evolve to similar endpoints[116]. When the paired substitutions in the same codon site result in different amino acids, we call it double divergence or divergent substitutions.

The divergence of the ancestors prior to a convergent event may also be of interest for more complex reasons. First, if the ancestors come from closely related species, the same wild population in the same species, or even replicate populations in the laboratory, the degree of convergence in response to the same selective pressure can be seen as a measure of mechanistic constraint. Convergence under these conditions may indicate that there are only a few easy ways to respond to that selective pressure. At the protein level, amino acid substitutions accumulate combinatorial epistatic effects as they diverge, leading to coevolution[117]. Such coevolution may alter the adaptive landscape but can also lead to decreasing levels of nearly neutral convergence (homoplasy) as proteins diverge. Second, the codon state of the ancestors can strongly affect the accessibility of the convergent state; many types of amino acid substitution are rare in part for this reason, and so convergence events involving one or more rare events may be a stronger indication that they are driven by selection rather than convergence involving common events. We discriminate between two classes of convergent events where the ancestral codon or amino acid states are different (discordant convergence) or the same (congruent convergence). We note that in using this terminology, we are avoiding the term "parallel evolution," which has rather ambiguous and muddled usage in the literature[116,118] and is sometimes applied to cases of similar or identical ancestral populations, species, biological systems, proteins, or amino acids.

Supplementary Text 3. New approaches to estimate the rate of molecular convergence. Among a variety of methods for conventional ω estimation[16,119], the so-called counting methods are most similar to our approach. First, ancestral codon sequences are estimated by the empirical Bayesian method devised in IQ-TREE[80], from which the probabilities of codon substitutions are calculated for each branch and site. The substitution probabilities are internally stored in multidimensional arrays designed for efficient processing of substitution probabilities (see Methods). Next, total probabilities of observed combinatorial substitutions (O_C) in a combination of two or more branches are obtained separately for nonsynonymous and synonymous substitutions (O_C^N and O_C^S, respectively) by deriving joint substitution probabilities with any, different, or specific states at the ancestral and the derived node of a branch (Supplementary Fig. 1c).

To obtain the total probabilities of expected combinatorial substitutions (E_C), we devised a method that utilizes codon substitution models similar to the previous report that leveraged amino acid substitution models in estimating excess convergence[18] (Supplementary Fig. 2a). A novel aspect of our approach is that it considers both nonsynonymous and synonymous substitutions. Codon transition probabilities are derived from a mechanistic or empirical codon substitution matrix, empirical codon equilibrium frequencies, branch length, site-wise substitution rates, and the ancestral states of the parent node. Using the expected codon states from this codon transition matrix, the joint probabilities of combinatorial substitutions are calculated as E_C^N and E_C^S, just as in the observed values (see Methods for details).

Finally, after accounting for different ranges of the synonymous and nonsynonymous rates of combinatorial substitutions (dS_C and dN_C, respectively, see Methods for the correction), a formula of the same form as that for calculating conventional ω was used to contrast the observed numbers of nonsynonymous and synonymous combinatorial substitutions with their respective expectations to derive ω_C by Equation 19. While ω_C is a general metric that can be calculated individually for different categories of combinatorial substitutions (Supplementary Fig. 1c), in this work, we consistently discuss the performance of $\omega_C^{\text{any}\rightarrow\text{spe}}$, which represents the rate of convergent substitutions, as it is among the most popularly analyzed types of combinatorial substitutions. Since we will be discussing convergent evolution in the rest of the current study, the superscript any → spe will be omitted unless otherwise mentioned.

There is another metric to analyze molecular convergence using both nonsynonymous and synonymous substitutions[120]. This metric, called P, contrasts the proportion of nonsynonymous convergence at nondegenerative nucleotide sites (their dN_P) and the proportion of synonymous convergence at four-fold degenerate nucleotide sites (their dS_P) in phylogenetic quartets. ω_C is distinct from P in many aspects, including the use of complete phylogenetic trees rather than decomposed quartets, the use of all codon sites regardless of their degree of codon degeneration, and the use of expected values based on a codon substitution model rather than the proportion of convergent substitutions.

Supplementary Text 4. Conventional approaches for estimating convergence rates. The metric R, for example, is intended to have an expectation of 1.0 under neutral evolution, but in practice is somewhat lower than 1.0, even when the tree and substitution model are correct and exactly match simulation conditions[18]. Using $R > 1.0$ as a criterion to identify convergence is thus in principle conservative for detecting convergence levels greater than fully neutral evolution. Furthermore, its accuracy depends on the accuracy of the phylogenetic tree in various aspects, e.g., neutral substitution model, tree topology, branch lengths, and reconstructed ancestral states. By contrast, the C/D comparison ratio, which compares convergence levels to double divergence events between branch pairs, is not strongly dependent on neutral substitution estimates[13,17]; however, it is dependent on the accuracy of the reconstructed tree compared to the true tree that applies. The C/D ratio may vary among proteins due to varying levels of constraint among proteins but is generally well below 1.0[17].

Divergent substitutions have the advantage of being linearly correlated with convergent substitutions[13,17], although, in C/D, the nature of comparing focal branch combinations to the others makes it difficult to identify certain evolutionary scenarios, such as widespread adaptive molecular convergence throughout the tree[18]. Expected numbers of convergent substitutions can be obtained from amino acid substitution models[18,26], such as the JTT model[121], in combination with observed amino acid frequencies in a protein, an amino acid site, or a group of amino acid sites categorized by the CAT model[122]. However, the difficulty in estimating equilibrium amino acid frequencies from a small number of proteins, especially when per-site frequencies are analyzed, hampers accurate expectations of convergent substitutions[18].

Both methods (utilizing divergent substitutions or expected convergence) successfully recover the pattern of diminishing convergence over time, a recently established evolutionary hallmark of proteins that evolve in the context of intramolecular epistasis[17,18,123]. However, false positives are difficult to eliminate due to errors in gene tree topologies caused by technical and biological factors, including incomplete lineage sorting, introgression, and within-locus recombination[9,14]. Regardless of whether the species tree or

individual gene trees are employed, this problem persists as a major source of false convergence in the analysis of genome-scale data.

Supplementary Text 5. Further evaluations of convergence metrics by simulations. To further check the robustness of ω_C, we analyzed simulated data under different settings. ω_C was stably estimated under a range of conventional ω values (0.1–5.0), indicating that ω_C successfully captures the change in substitution profiles but not the change in the rate of protein evolution (Supplementary Fig. 4a). A robust estimation was generally achieved even if the codon substitution model was mis-specified in the ancestral reconstruction step (Supplementary Fig. 4b). One exception was the use of unrealistically simple reconstruction models (MG and GY), in which the variances of dN_C and ω_C increased while the median did not change greatly. Therefore, care should be taken when a simple model is used. ω_C was robust against other factors, as mentioned in the main text (Supplementary Fig. 4c–g).

Supplementary Text 6. Signature of intramolecular epistasis in empirical convergence. In the known examples of adaptive protein convergence, we found that the rate of concordant convergence ($\omega_C^{\mathrm{spe}\to\mathrm{spe}}$) is significantly higher than that of discordant convergence ($\omega_C^{\mathrm{dif}\to\mathrm{spe}}$), with the largest contribution to the χ^2 statistic coming from depleted nonsynonymous substitutions in discordant convergence (Supplementary Fig. 5j–k, *P*-value is shown in the plot). Such a pattern was not detected in the simulated adaptive convergence (Supplementary Fig. 4a). The simulated codon sequence evolution assumes independence between sites; therefore, intramolecular epistasis is ignored. In the presence of epistasis between amino acid sites, a substitution at one site will change the substitution profiles of other coupled sites[124], and subsequent substitutions in the coupled sites entrench the original site[117,125,126]. This means that epistasis makes it difficult to replace different ancestral amino acids with the same derived amino acid, even in homologous sites in the same protein (Supplementary Fig. 5l). Thus, intramolecular epistasis can be a source of the different rates between concordant and discordant convergence.

Supplementary Text 7. Temporal variation of convergence rates. The probability of protein convergence decreases over time, with intramolecular epistasis among amino acid residues considered to be a primary biological source of such an evolutionary pattern[17,18,117]. Indeed, over a long timescale, the environment around any given focal site changes through substitutions at other amino acid sites, thus altering which amino acid state at the focal site is suitable to maintain structure and function[117,124] (Supplementary Fig. 5l). However, gene tree discordance due to biological and technical causes, including tree inference error, incomplete lineage sorting, introgression, HGT, and intralocus recombination, can create a false convergence signal that similarly decreases with the time since branches separated[9,14] (Fig. 1a and Supplementary Fig. 1a). While the analysis of the mitochondrial genome[17] would not have been confounded by recombination-mediated mechanisms, other factors would have as great an influence as for nucleus-encoded genes. Nevertheless, all of the above problems would produce false convergence signals equally in synonymous and nonsynonymous substitutions via errors in the phylogenetic tree topology; therefore, ω_C should be a natural candidate to unbiasedly evaluate whether convergence rates in nucleus-encoded genes also decrease with time.

With the 21-vertebrate genome dataset, we analyzed 2,349,515 branch pairs with at least one synonymous and nonsynonymous convergence (i.e., $O_C^N \geq 1.0$ and $O_C^S \geq 1.0$). In all metrics (C/D, dN_C, and ω_C), protein convergence rates clearly decreased over time (approximated by inter-branch genetic distance) (Fig. 2b). Notably, we observed no such pattern for the rate of synonymous convergence (dS_C), making it more likely that the diminishing protein convergence is caused by evolutionarily selected mechanisms[17,18].

To further characterize rate decreases over time, we took advantage of the ability to apply ω_C to a variety of combinatorial substitutions. We asked whether the rate decrease is specific to convergence by performing the same analysis for other categories of combinatorial substitutions (Supplementary Fig. 1c). Notably, the rate of double divergence decreased over time in a manner similar to the decrease in

convergence (Supplementary Fig. 8b). The sum of double divergence and convergence corresponds to paired substitutions (Supplementary Fig. 1c), the rate of which also decreased over time (Supplementary Fig. 8c). These results suggest two possibilities. One result is that epistatic changes from neighboring amino acid residues impose constraints on not only to which amino acid state a site tends to substitute (i.e., site-specific substitution profile), but also on which amino acid sites tend to substitute (i.e., site-specific substitution rate). The alternative (not necessarily exclusive) possibility is that doubly divergent events are decreasing because the rate of convergence to similar but not identical amino acids decreases just as the rate of convergence to identical amino acids decreases. In either case, this effect may be important to account for in analyses of adaptation.

Thus, the pattern of diminishing convergence remains a clear trend in recombining nucleus-encoded genes, even after correcting for the rate of synonymous convergence, and therefore is consistent with the action of intramolecular epistasis (Supplementary Fig. 5l).

Supplementary Text 8. Gene duplication decreases convergence rates. Gene duplication generates new genetic building blocks[127] and elevates the rate of protein evolution[44]. However, it remains unknown whether substitution profile changes influence convergence rates following gene duplication. Convergent substitutions in duplicates may indicate convergent functional changes in independently duplicated genes, and our genome-scale dataset contains 90,028 duplication events, providing an excellent opportunity to address this question. If independent duplications in a family of genes tend to result in mutually similar derived pairs of proteins, the convergence rate should increase. Conversely, if the new proteins tend to move into a divergent sequence space in which they do not overlap, gene duplication would not increase convergence and may even decrease it. Accelerated non-adaptive change might not change the convergence rate if gene duplication only causes an increase in the rate of protein evolution without changing the substitution profiles. To distinguish these possibilities, we compared the convergence rates of branch pairs after two separate speciation (SS) events and branch pairs after two independent gene duplications (DD) (Fig. 2c, Supplementary Text 9, and Supplementary Fig. 8d,e). It should be noted that this analysis does not compare orthologs versus paralogs but assesses the effect of gene duplication, relative to the baseline mode of protein sequence evolution after speciation. Strikingly, gene duplication significantly decreased convergence rates ($P \approx 0$, $W = 23.0$, as determined by a two-sided Brunner–Munzel test; Fig. 2c). Again, the trend was evident in nonsynonymous convergence (dN_C) but not in synonymous convergence (dS_C), implying a relaxation in site-specific constraints or adaptive divergence in the duplicates. Notably, the effect of gene duplication was stronger in closely related branch pairs (i.e., smaller bin numbers in Fig. 2c), and the ω_C distributions became progressively indistinguishable between SS and DD pairs with increasing inter-branch distance. The immediate drop of the convergence probability was consistent with the idea that gene duplication allows the new gene copies to explore a new sequence space, potentially involving natural selection. We note that this is an averaged trend across genes and does not exclude possible adaptive convergence in some genes. However, it is likely that such convergence, if it does exist, is masked by the opposing, predominant signal of relaxed or divergent constraints.

Supplementary Text 9. Potential artifacts arising from falsely placed gene duplications and false gene grouping. It is noteworthy that the DD branch pairs show anomalously high synonymous convergence rates (dS_C) in the smallest bin of genetic distance (bin 1 in Fig. 2c). This observation is probably due to the difficulty of locating gene duplication events in the phylogenetic tree, especially when sequences are not sufficiently diverged and lead to an extremely short branch length. Consistent with this idea, small genetic distances were associated with low branch supports in the DD branch pairs (Supplementary Fig. 8d). Additionally, we sometimes observed anomalously high synonymous convergence rate (dS_C) in extremely distant branch pairs, which can be attributed to an incorrect grouping of different gene families. Although orthogroup inference has dramatically improved in accuracy in recent years[32,100], it does not completely eliminate false groupings. In line with this idea, orthogroups that encompass extremely large genetic distances tend to contain multiple sets of genes that have clearly non-homologous sets of protein domains

(Supplementary Fig. 8e; Supplementary Dataset[94] for orthogroups with total branch distance greater than 15 nucleotide substitutions per nucleotide site). These examples illustrate how various aspects of phylogenetic analysis can generate false patterns of convergence that are successfully captured by dS_C and corrected for in ω_C.

Supplementary Text 10. Factors affecting the number of branch combinations in genome-scale analysis. The number of gene branch combinations mapped to a species branch combination depends on a variety of factors, and therefore the number of detected convergence per species branch pair can vary non-adaptively. Overall, terminal branches in the species tree tended to involve larger numbers of gene branch combinations than internal branches (Supplementary Fig. 10a). This is likely because the more terminal the species branches, the more gene branches for comparison due to the accumulation of branches generated by historical gene duplications. Another factor that may explain the differences between internal and terminal branches is branch lengths in the species tree, which should be correlated with the number of gene duplications under a relatively constant gene duplication rate. Indeed, the product of species branch lengths showed a moderate correlation with the number of gene branch combinations (Spearman's ρ = 0.283, Supplementary Fig. 10a). Inheriting this heterogeneity, the number of convergent branch pairs also varies greatly among species branch pairs (Supplementary Fig. 10b), with Spearman's correlation coefficient as high as 0.745 (Supplementary Fig. 10c). The terminal branch connected to *Danio rerio* showed an unusually high number of gene branch combinations (diagonal elements in Supplementary Fig. 10a,b). This feature may partly be explained by the number of annotated genes in this species, which was the highest among analyzed genomes (Supplementary Fig. 10d). In large orthogroups dominated by *Danio* genes, a large number of branch pairs should be generated for the comparison of two *Danio* gene lineages.

Supplementary Text 11. Examples of joint expression–protein convergence. Compelling examples included members of aldo-keto reductase family 1 (AKR1), which play essential roles in steroid metabolism[128]. The OU analysis revealed that AKR1 acquired preferential expression in the ovary after repeated lineage-specific duplications in rabbits and mice (*Mus musculus*) (Fig. 3d). Among the paired substitutions in the two lineages, F129I (convergence) and F306A/V (double divergence) located to the positions that delineate the steroid-binding cavity (Fig. 3d). At residue 306, the size of the amino acid was shown by targeted mutagenesis to be important for catalytic promiscuity in rabbits[129]. Similarly, D224C/E (double divergence) occurred in a loop that contributes to substrate specificity[129]. These results suggest that the phenotypic change related to substrate specificity might have occurred not only in rabbits but also in mice and underscore how F129I, together with the other two convergence cases (N11S and T/S289P, Supplementary Fig. 11a), should be a major target for future characterization.

Similarly, nudix hydrolase 16-like 1 (NUDT16L1, also known as Tudor-interacting repair regulator [TIRR]), which is involved in cell migration[130] and whose encoded protein binds to RNA and P53-binding protein 1 (53BP1)[131], showed lineage-specific duplications in chinchillas (*Chinchilla lanigera*) and another rodent lineage connected to mice and rats (*Rattus norvegicus*) (Fig. 3e). The duplication events were followed by convergent regime shifts that resulted in testis-specific expression. The expression evolution was coupled with convergent substitutions in the protein sites corresponding to the substrate-binding pocket of the de-ADP-ribosylating homolog NUDT16[132,133]. Protein convergence linked to testis-specific expression was also observed in myeloid-associated differentiation marker (MYADM), which encodes a transmembrane protein that localizes to membrane rafts[134], regulates eosinophil apoptosis through binding to Surfactant protein A (SP-A)[135], and participates in cell proliferation and migration[136]. This orthogroup showed joint convergence in two pairs of branches, in both of which the convergent amino acid substitutions were almost entirely confined to one side of the transmembrane domains (Fig. 3f), suggesting altered interactions with other molecules through this portion of the protein.

Supplementary Text 12. Higher-order convergence in the 21 animal genomes. We developed a heuristic approach similar to a method used for the detection of higher-order transcription factor combinations in gene

regulation[137]. This method can also be considered a type of greedy algorithm because it determines the search range for higher-order branch combinations based on the convergence rate of lower-order branch combinations. To further characterize the heuristic search of highly-repetitive convergence, we again analyzed the 21 animal genomes. The same threshold as in the analysis of PEPC ($\omega_C \geq 5.0$ and $O_C^N \geq 2.0$) was applied to search branch combinations up to $K = 10$ (i.e., convergence among 10 branches). Up to $K = 3$, the numbers of convergent branch combinations were two orders of magnitude less than the numbers of analyzed combinations, but thereafter, the difference was drastically reduced, indicating an efficient search of branch combination space (Supplementary Fig. 13a and Supplementary Table 9).

At $K = 10$, only two out of 16,724 orthogroups were detected to contain convergent combinations. Upon closer examination, one of them (OG0000136, encoding Glutamate receptors and containing 742 out of 746 detected combinations at $K = 10$) was found to be a likely artifact due to different splicing variants being inconsistently included in the representative gene set for each species (Supplementary Fig. 13b). In the animal genome analysis, we selected the longest transcript among splicing variants according to common practice[44], and this operation seems to create the artifacts. A characteristic feature of this artifact is that many combinatorial substitutions are concentrated to a narrow window of the protein sequence (Supplementary Fig. 13b). Protein convergence at $K = 2$ shown in Supplementary Fig. 11a did not show such a feature, and therefore this problem may be pronounced particularly when analyzing higher-order convergence. We expect synonymous convergence to cancel out the false signal in many cases, but in the cases where synonymous substitutions did not happen or are largely lost, the artifacts are not completely excluded from the results of genome-scale analyses.

The other detected orthogroup at $K = 10$ (OG0000062, encoding Protocadherin beta) did not show the signature of false convergence due to inconsistently represented alternative transcripts (Supplementary Fig. 13c). Only two lineages were involved in the four sets of 10 detected branches: pigs and the lineage connected to mice and rats (Supplementary Fig. 13d). At such a high-order convergence, no synonymous convergence was not detected at all, so ω_C diverged to infinity in four detected branch combinations. Although alternative mechanisms such as gene conversion may be involved, this orthogroup may represent a case of biologically generated highly-repetitive convergence, with a possibility of a highly coevolving pair of amino acid sites in a unit of the extracellular cadherin repeats (Supplementary Fig. 13e).

In the two amino acid sites, it appears that the same substitutions occurred outside of the 10 lineages, but they were not detected with the threshold we used (Supplementary Fig. 13d). Although only 21 species were included in this genome-scale analysis, a larger set of genomes will enable the detection of higher-order molecular convergence that correlates well with phenotypes, as in the case of PEPC. Alternatively, it may be possible to detect weak convergent signals by concatenating genes in similar functional categories[138]. A limitation of such an approach is that groups of genes containing lineage-specific duplications or losses cannot be analyzed.

Supplementary Text 13. Recommended usage of ω_C and O_C^N. It is useful to provide some clarification of our recommended usage of the ω_C statistic, particularly because in this manuscript we used different thresholds, depending on the context, to characterize ω_C values that indicate genes of interest. In the simulation analyses, we used the neutral simulations (1,000 replications) to define false positives with the 95th percentile threshold in test simulations (Fig. 1d). Because these are simulations, we know the number of true positives, and can also calculate the true positive rate distribution across simulations (Supplementary Fig. 3). While this provides a useful theoretical guide to the behavior of the statistic, it should be recognized that sites were independent of each other in these simulations. In real proteins, epistasis may affect the generation of both true and false positives. Thermodynamic simulations of protein evolution[117] may be able to overcome this problem but would require a large number of costly computational simulations, and still, the improvement in predictive value for real data would not be certain and would require extensive validation that is beyond the scope of this paper. Permutations are an alternative approach to obtaining false-positive convergence estimates, but we caution that it is not certain how substitutions should be randomized in the context of epistasis and among sites with varying constraints and substitution

rates; again, any such approach would make uncertain improvements and would require extensive validation. To manage this uncertainty in our real data analysis, we compared the top 1% of values among methods (a rank-based threshold) to allow a fair comparison of different convergence metrics within the same dataset (i.e., C/D, dN_C, or ω_C). In genome-scale analyses, such rank-based thresholds can extract the most promising convergent branch combinations among a large number of observations. For particular gene families or specific lineage combinations, the number of observations is often small and it is useful to choose a reasonable threshold based on our analyses so far. For the animal genome analysis, we used a threshold of ω_C greater than 3.0 that corresponds to the 92nd percentile of all analyzed branch pairs with more than three nonsynonymous convergence ($O_C^N \geq 3.0$), while in the higher-order analysis of PEPC we employed a more stringent threshold ($\omega_C > 5.0$) to control for the combinatorial explosion. The threshold setting for ω_C should be considered and potentially adjusted based on the above caveats and the needs of a given research project, but ultimately the utility of convergence analysis will depend on the validated utility of these predictions across a variety of biological contexts, and we recommend these thresholds as reasonable standardized starting points based on our analyses.

Protein convergence has attracted a great deal of attention for its potential to associate long-term genotypic variation with phenotypic change, from its first discovery[24], subsequent theoretical development[13,26], the first claim of genome-wide detection[115], to recent findings that highlighted epistatic effects[17,18,117,123] and technical difficulties[9–12,14]. Other types of convergence at the molecular level beyond amino acid substitutions have also been considered, including convergent shifts of site-wise substitution profiles[139], convergent shifts of evolutionary rates (i.e., number of substitutions per time regardless of the amino acid state or substitution profile)[140], convergent rate shifts of noncoding elements[141], convergent gene losses[142,143], convergent losses of noncoding elements[144], and functional enrichments of convergently evolved loci[138]. Using transcriptome amalgamation, which integrates multi-species gene expression data in a comparable manner[44], we developed a means to detect convergence in gene expression levels and to correlate the obtained results with protein convergence rates. Further integration of these methods will allow us to examine how well convergent patterns correlate across multiple hierarchies of biological organizations. Such analysis will provide a quantitative perspective of the extent to which evolution at one hierarchical level causes predictable changes in another.

Supplementary Text 14. Genome editing as a means to evaluate the mutational effects of molecular convergence. The rapid development of genome editing technologies with CRISPR/Cas-based systems[145,146] provides a means to test the effect of mutations on *in vivo* phenotypes using targeted mutagenesis. This approach can help us understand important biological processes, for example, for livestock and crop enhancements. However, because of the massive mutations accumulated in the lineage of interest, a key challenge is the efficient identification of important mutations, and even more so for combinations of mutations because mutational effects are often dependent on genetic background[147]. Convergent evolution, which can be seen as replicated experiments by nature, has the potential to solve this problem. Convergent mutations that arise in different lineages are likely to have stronger effects and depend less on the genetic background than mutations that were not convergent under the same physiological or phenotypic adaptive pressure, and such mutations and the genes that carry them are thus promising candidates to achieve desired phenotypes. One successful example is the toxin resistance conferred to an engineered fruit fly strain, "monarch fly," which harbors convergent amino acid substitutions, also found in monarch butterflies, in its sodium pump ATPalpha1[148,149]. As such, adaptive molecular convergence discovered by our method could be experimentally verified while utilizing genome editing.

Supplementary Text 15. Use of posterior probabilities of ancestral states for the inference of substitutions. To estimate the posterior probabilities of substitutions, we sum over the posterior probabilities of ancestral states. In this way, we circumvent a computationally expensive step employed in previous reports to handle individual Markov chain Monte Carlo (MCMC) samples separately[17,27]. However, since the posterior probabilities are not independent for each node of a phylogenetic tree, this approximation comes

at the expense of accuracy in estimating substitution probabilities. In the analysis of amino acid sequences, it is difficult to exclude such a bias. In contrast, in our method, this bias appears in both nonsynonymous and synonymous substitutions and is likely to be canceled out when calculating ω_C, the ratio of their convergence rates. To assess the impact of summing over the ancestral state posteriors, we reanalyzed the vertebrate genome dataset with the CSUBST option --ml_anc to binarize the posterior probabilities in the three-dimensional arrays with the size of $M \times L \times 61$ (see Methods). This operation corresponds to the uniformization between MCMC samples, and the substitution probabilities are binarized accordingly. In this setting, we reproduced the analysis shown in Fig. 2b. Although the temporal trends were consistent, the convergence metrics, especially dN_C and dS_C, were slightly higher than those in Fig. 2b (i.e., more conservative without binarization) (Supplementary Fig. 14). Importantly, such a shift was less evident in ω_C, as expected. These observations led us to adopt the approximation of substitution probabilities in the ω_C calculation to take advantage of computational speed-up.

Supplementary Text 16. Protein size–dependent change in convergence rates. The genome-scale analysis of vertebrate genes allowed us to correlate various protein properties with convergence rates. In the course of analysis, we found that protein sizes negatively correlate with convergence rates (ρ = −0.11 with C/D and ρ = −0.11 with dN_C; Supplementary Fig. 16a). Unlike the temporal variation, it is difficult to explain this trend with epistasis because larger proteins should have more epistatic interactions that increase convergence probability[17,18,150]. In addition, protein size does not correlate with genetic distance (ρ = 0.01; Supplementary Fig. 16b), confirming that confounding is negligible. A similar trend in synonymous convergence rate (ρ = −0.07 with dS_C) suggests that, unlike the temporal variation (Fig. 2b), the pattern is largely nonbiological and perhaps created by the uncertainty caused by the small number of codon substitutions in small genes. As the trend is consistently observed in nonsynonymous and synonymous convergence rates, ω_C was relatively stable over protein size (ρ = −0.06), further demonstrating its robustness against artifacts.

Supplementary Text 17. Remarks on empirical datasets. For benchmarking, we collected known examples of molecular convergence associated with phenotypes. While we followed the same taxon sampling as in the original reports (cited in the main text), further additions and scrutiny of taxa allowed us to find previously unappreciated features in some datasets.

The convergence of mitochondrial proteins between snakes and lizards of the Agamidae family was reported previously[13]. In our mitochondrial genome dataset, a massive burst of amino acid convergence was found between snakes and Acrodonta, the lineage consisting of not only Agamidae but also Chamaeleonidae. This detail was not in the previous report because Chamaeleonidae were not available at the time to be included in the phylogenetic analysis.

Improved phylogenetic resolution is known to increase the specificity of convergent site detection[15]. In carnivorous plants, several amino acid substitutions were reported previously in digestive enzymes[27]. With additional plant genomes (Supplementary Table 10), the candidate convergent substitutions were narrowed down in this study to smaller numbers of substitutions that correlated more tightly in the phylogenetic placement with the evolution of carnivory. One of the convergent substitutions found in both the previous report and this study is located at a substrate-binding site in the glycoside hydrolase family 19 (GH19) chitinases (Supplementary Fig. 5f). Double divergence was found in a substrate-binding site of PAPs (Supplementary Fig. 5g).

Supplementary Tables

Supplementary Table 1. Methods to detect convergent signatures of protein sequences. (separate file)

Supplementary Table 2. Parameter settings for the simulated molecular evolution. (separate file)

Supplementary Table 3. Summary of empirically validated protein convergence. (separate file)

Supplementary Table 4. Convergence statistics in empirically validated protein convergence. (separate file)

Supplementary Table 5. List of branch pairs with herbivory-associated protein convergence. (separate file)

Supplementary Table 6. List of branch pairs where simultaneous convergence of gene expression and protein sequences is detected. (separate file)

Supplementary Table 7. Enrichment of joint expression–protein convergence associated with different tissues. (separate file)

Supplementary Table 8. Time required for the analysis of higher-order convergence in PEPC. (separate file)

Supplementary Table 9. Orthogroups and branch combinations detected in the genome-scale analysis of higher-order convergence. (separate file)

Supplementary Table 10. Genome and transcriptome data. (separate file)

Supplementary Figures

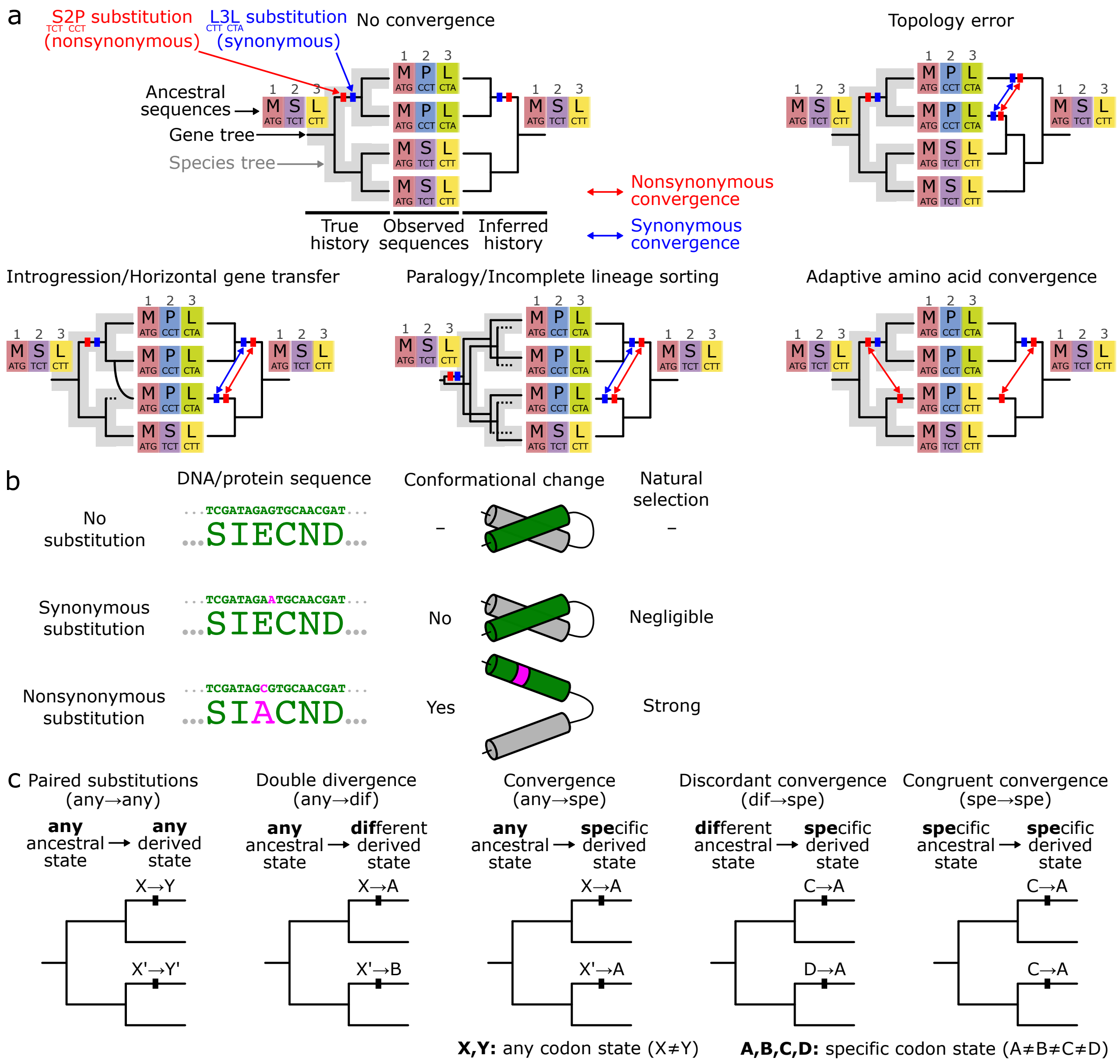

Supplementary Figure 1. Types of substitution and their relationships to evolutionary patterns. (a) Errors in tree topology lead to false convergence. No convergence is detected as long as the phylogenetic tree is correctly inferred, while errors in the tree topology can lead to spurious convergence. Even if the species tree is correctly inferred, there can still be spurious convergence if introgression or horizontal gene transfer (HGT) has occurred. A similar situation can arise from paralogy and incomplete lineage sorting. While the above technical and biological factors alter the inference of both nonsynonymous and synonymous substitutions, adaptive convergence should involve an increased rate of nonsynonymous convergence without changing synonymous convergence. (**b**) The relationship between the type of substitution, protein conformation, and natural selection. (**c**) Combinatorial substitutions with evolutionary importance. A pair of substitutions at the same site in two lineages are annotated on branches (ancestral→derived). X and Y indicate any codon state, and A, B, C, and D denote specific codon states.

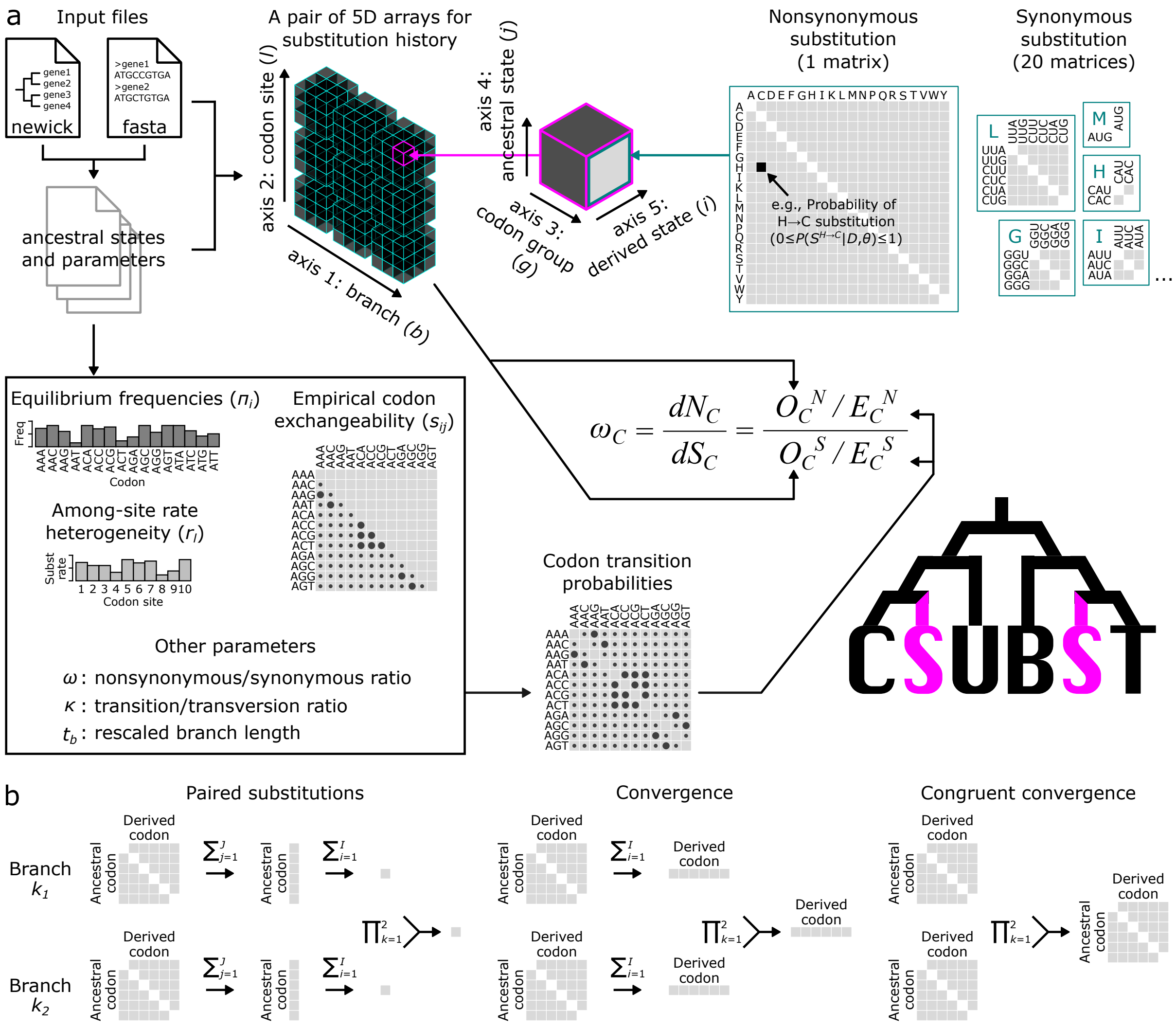

Supplementary Figure 2. Overview of the method. (**a**) Flow of data in CSUBST. (**b**) Array operations for deriving the probabilities of combinatorial substitutions.

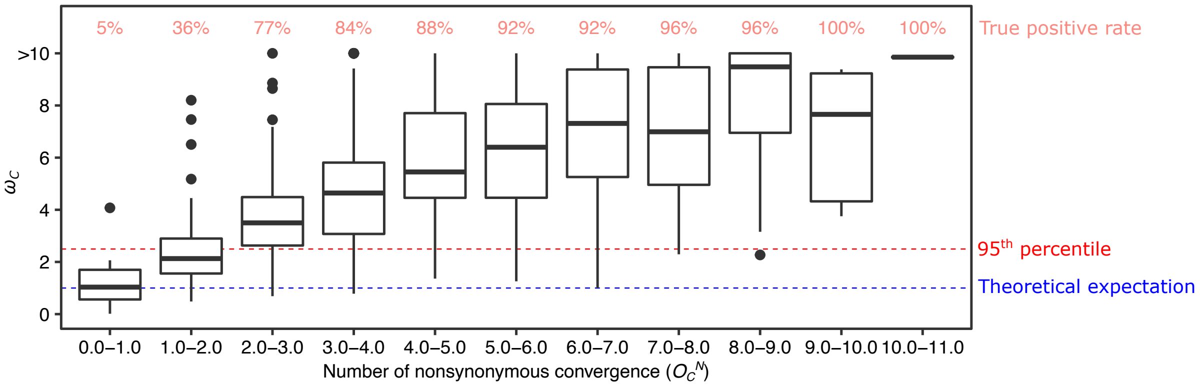

Supplementary Figure 3. The true positive rate increases with the number of convergent substitutions. Simulated data analyzed were from the Convergent scenario of Fig. 1d (N = 1,000 simulations). Boxplot elements are defined as follows: center line, median; box limits, upper and lower quartiles; whiskers, 1.5 × interquartile range.

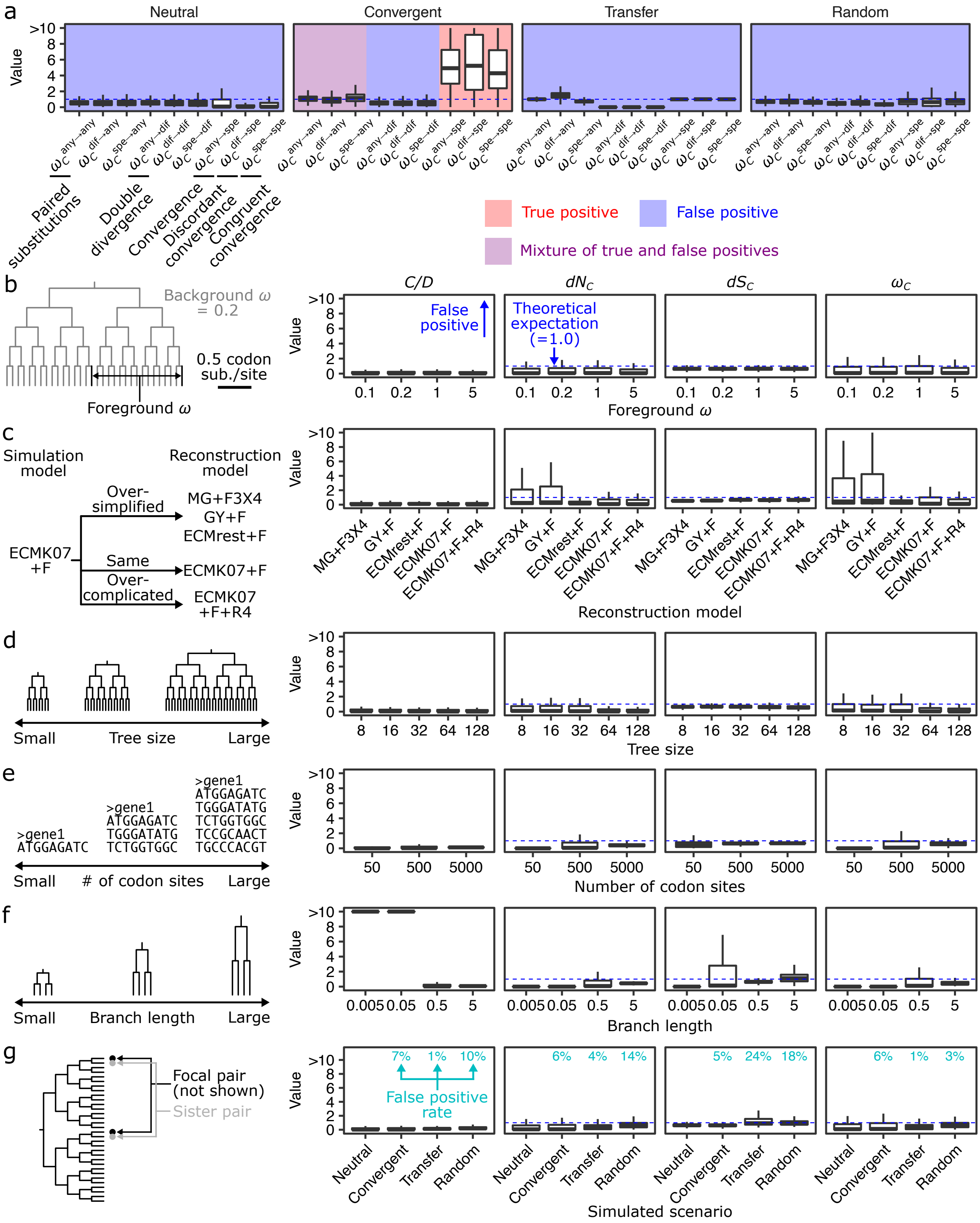

Supplementary Figure 4. **Robustness of convergence metrics under simulated conditions.** (**a**) Comparison of the complete set of ω_C variants. There are nine ω_C variants, of which three are associated with convergence: $\omega_C^{\text{any}\rightarrow\text{spe}}$, $\omega_C^{\text{dif}\rightarrow\text{spe}}$, and $\omega_C^{\text{spe}\rightarrow\text{spe}}$. Boxplot elements are defined as follows: center line, median; box limits, upper and lower quartiles; whiskers, 1.5 × interquartile range. N = 1,000 simulations. (**b**) Conventional ω values. According to the value of ω, the mode of protein evolution can be categorized into purifying selection ($\omega < 1$), evolution without constraint ($\omega = 1$), and adaptive evolution ($\omega > 1$). The examined parameters are illustrated on the left in **b**–**g**. If no changes are indicated, the parameters of the simulations are the same as in the Neutral scenario in Fig. 1c,d. To the right, each box plot corresponds to the results of 1,000 simulations. Dashed lines indicate the theoretical expectation (=1.0) except for C/D, for

which no theoretical expectation is available. (**c**) Model misspecifications. The following base models were analyzed: MG[82], GY[83], ECMrest[74], and ECMK07[74]. (**d**) Tree sizes. (**e**) Number of codon sites. (**f**) Branch lengths. When the branch length equals 1, an average of one substitution occurs per codon site. (**g**) Sister branches. The pairs of branches sister to focal branches in Fig. 1c,d were analyzed.

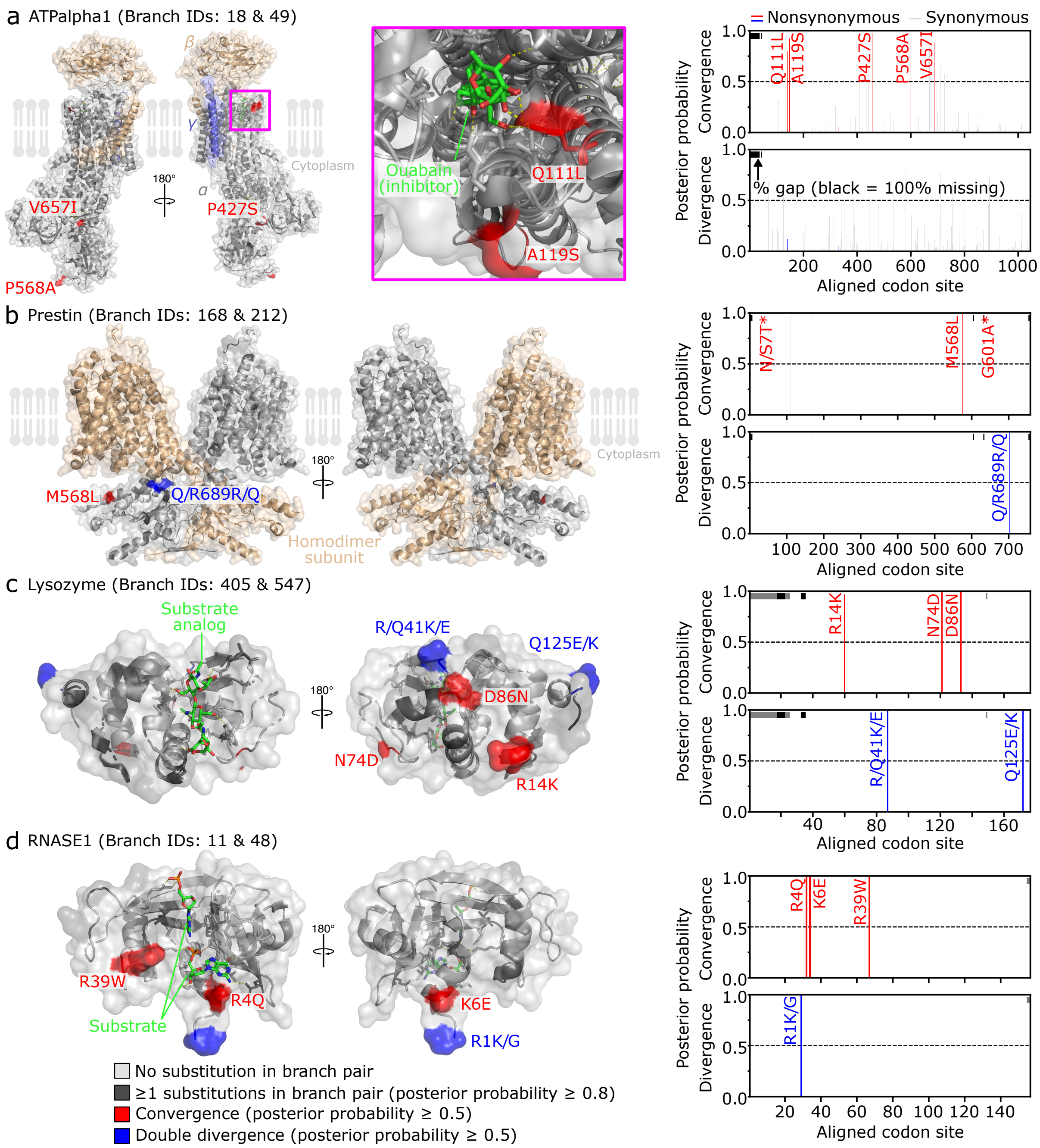

Supplementary Figure 5. Convergence metrics in genes associated with phenotypic convergence. (**a**–**h**) Mapping of combinatorial substitutions to the protein structures of ATPalpha1 (**a**, PDB ID: 4HYT), Prestin (**b**, 7LGU), Lysozyme (**c**, 9LYZ), RNASE1 (**d**, 2QCA), RNase T2 (**e**, 1VCZ), GH19 chitinase (**f**, 4IJ4), PAP (**g**, 6GIZ), and PEPC (**h**, 6MGI). The surface representation of the protein is overlaid with a cartoon representation. Convergent and divergent amino acid loci are highlighted in red and blue, respectively. Substrates and their analogs are shown as green sticks. Side chains forming the substrate-binding site are also shown as sticks. Note that these are the side chains in the protein from databases, so amino acid substitutions in the convergent lineages may result in distinct structures and arrangements. The probability of combinatorial substitution for each codon site is shown to the right. Asterisks indicate sites that are not included in the PDB protein structure. Site number 0 indicates no homologous site in the PDB protein structure. A representative branch pair is shown when three or more convergent lineages exist. (**i**) Known examples of protein convergences and HGTs were analyzed with C/D, dN_C, dS_C, and ω_C. Encoded proteins, associated traits, and numbers of sequences and codon sites are provided along the y-axis labels. The images to the right depict the organisms representative of the focal lineages. Points correspond to

individual pairs of branches in the gene tree (shown in Supplementary Fig. 6 and Supplementary Fig. 7). The photograph of *Alloteropsis semialata* is licensed under CC BY 4.0 (https://creativecommons.org/licenses/by/4.0/) by Alexey Yakovlev. (**j**) Comparison of the complete set of ω_C variants. Points correspond to individual gene trees. Horizontal bars indicate median values. (**k**) χ^2 test comparing the number of combinatorial substitutions associated with concordant convergence and those associated with discordant convergence. The number of combinatorial substitutions in all focal branch pairs of known protein convergence was summed. Circle sizes and colors indicate the relative contribution to the χ^2 statistic. (**l**) Schematic representation of the relationships between intra-molecular epistasis and the rates of convergence. As the inter-branch distance increases, the local environment around the amino acid site changes in the protein structure, leading to a change in the propensity of amino acid substitutions[17,117].

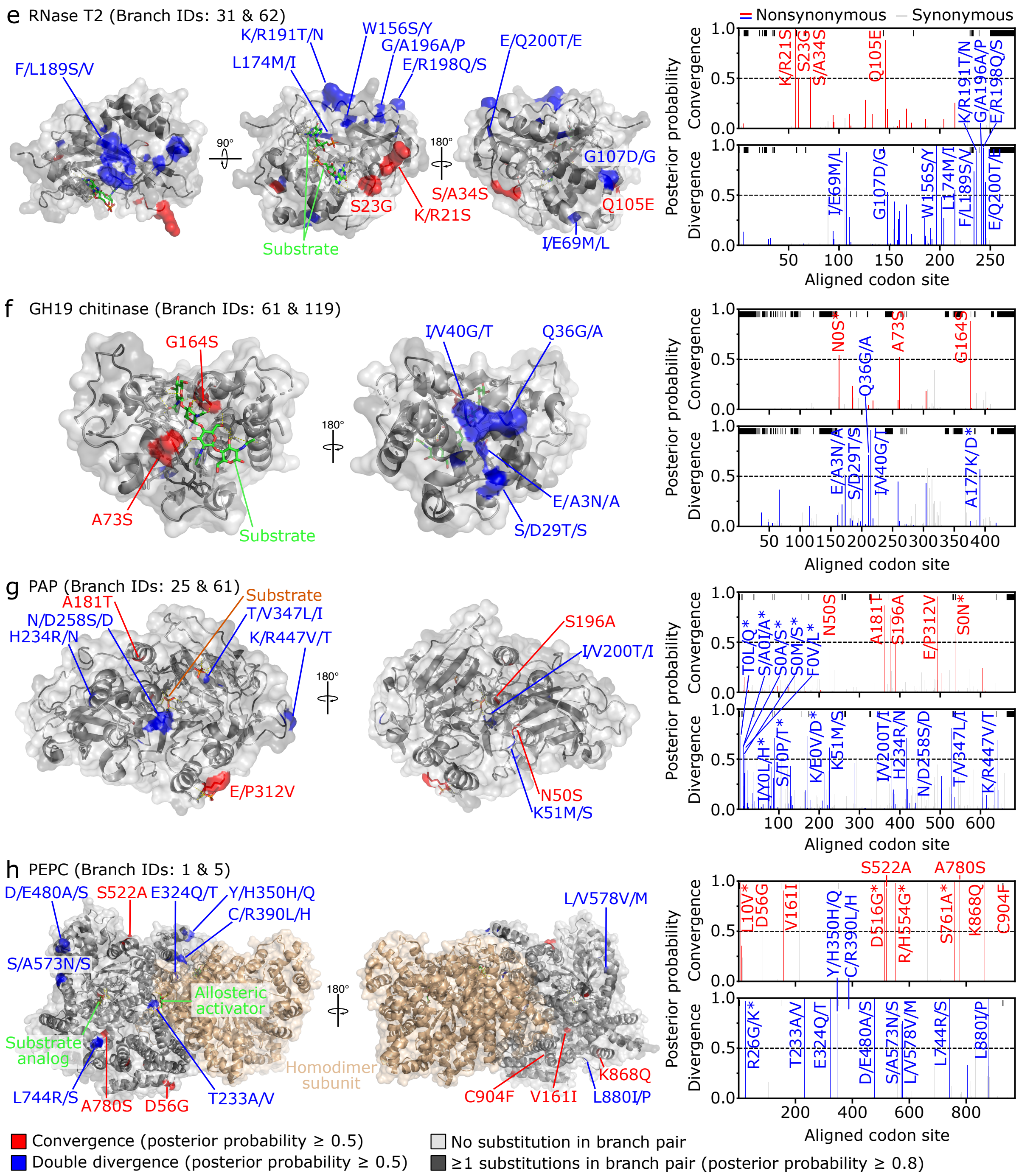

Supplementary Fig. 5 (continued)

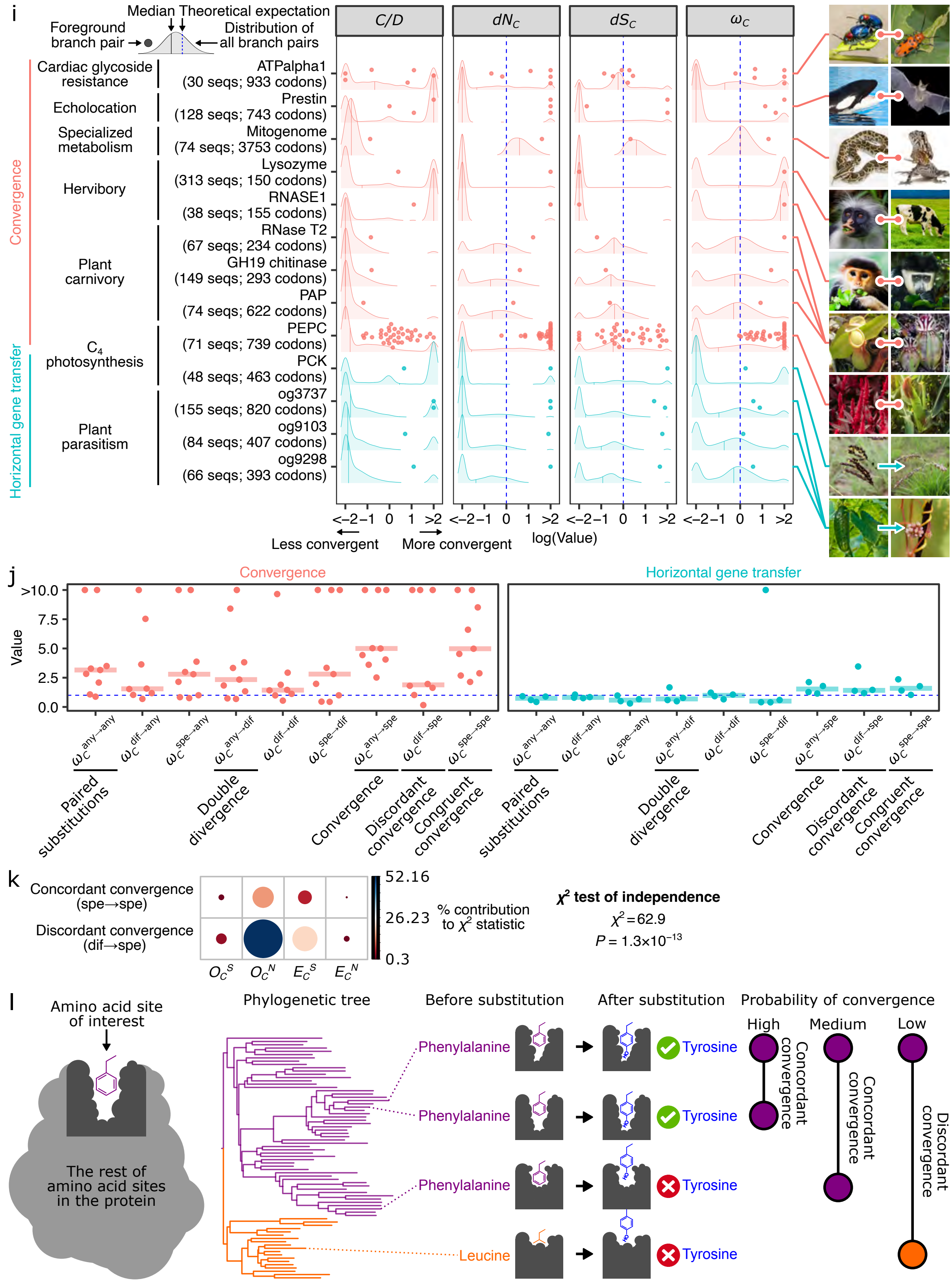

Supplementary Fig. 5 (continued)

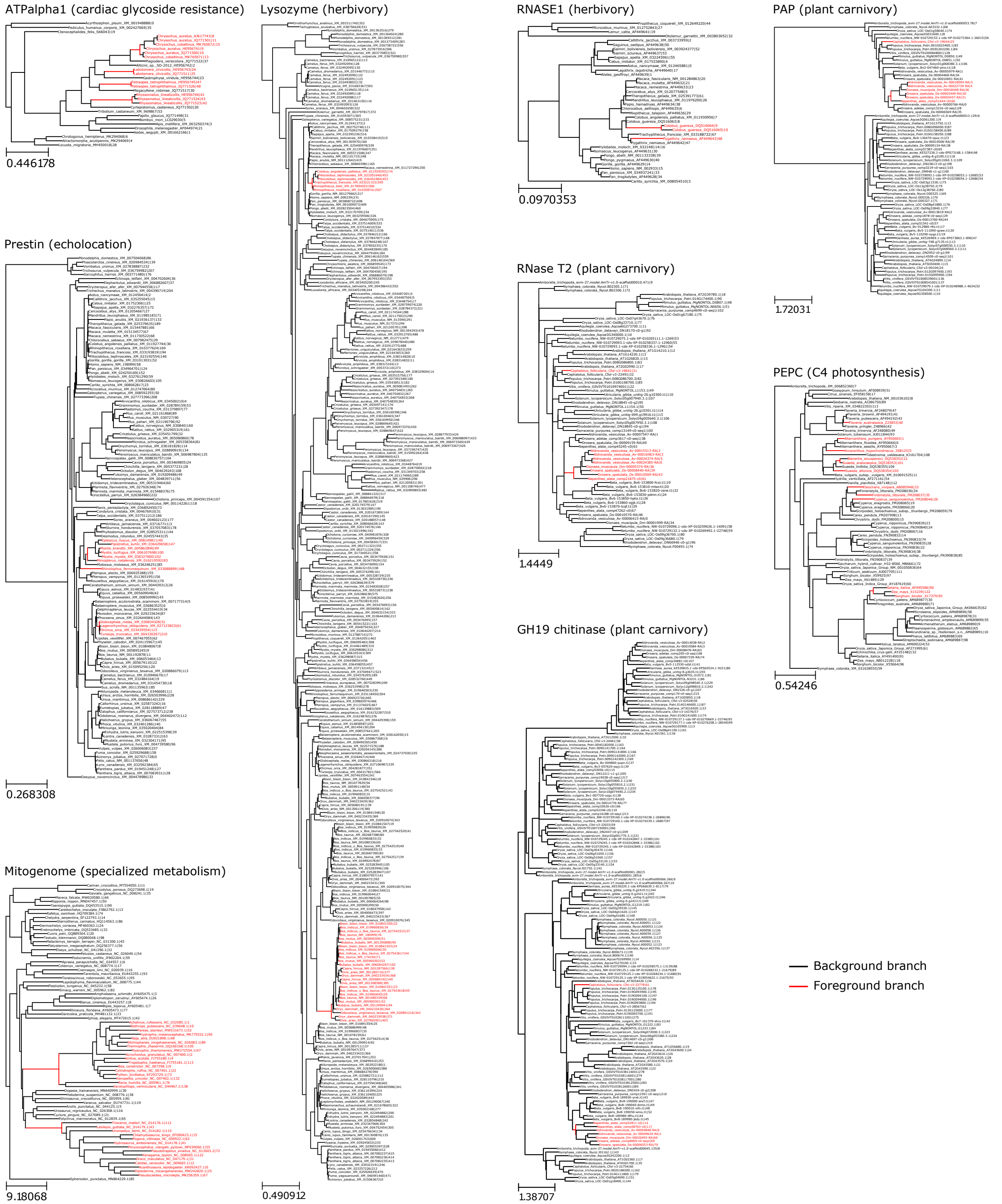

Supplementary Figure 6. Maximum-likelihood phylogenetic trees for the reported cases of convergent evolution. Scale bars indicate substitutions per nucleotide site. Red indicates focal branches (Fig. 1e).

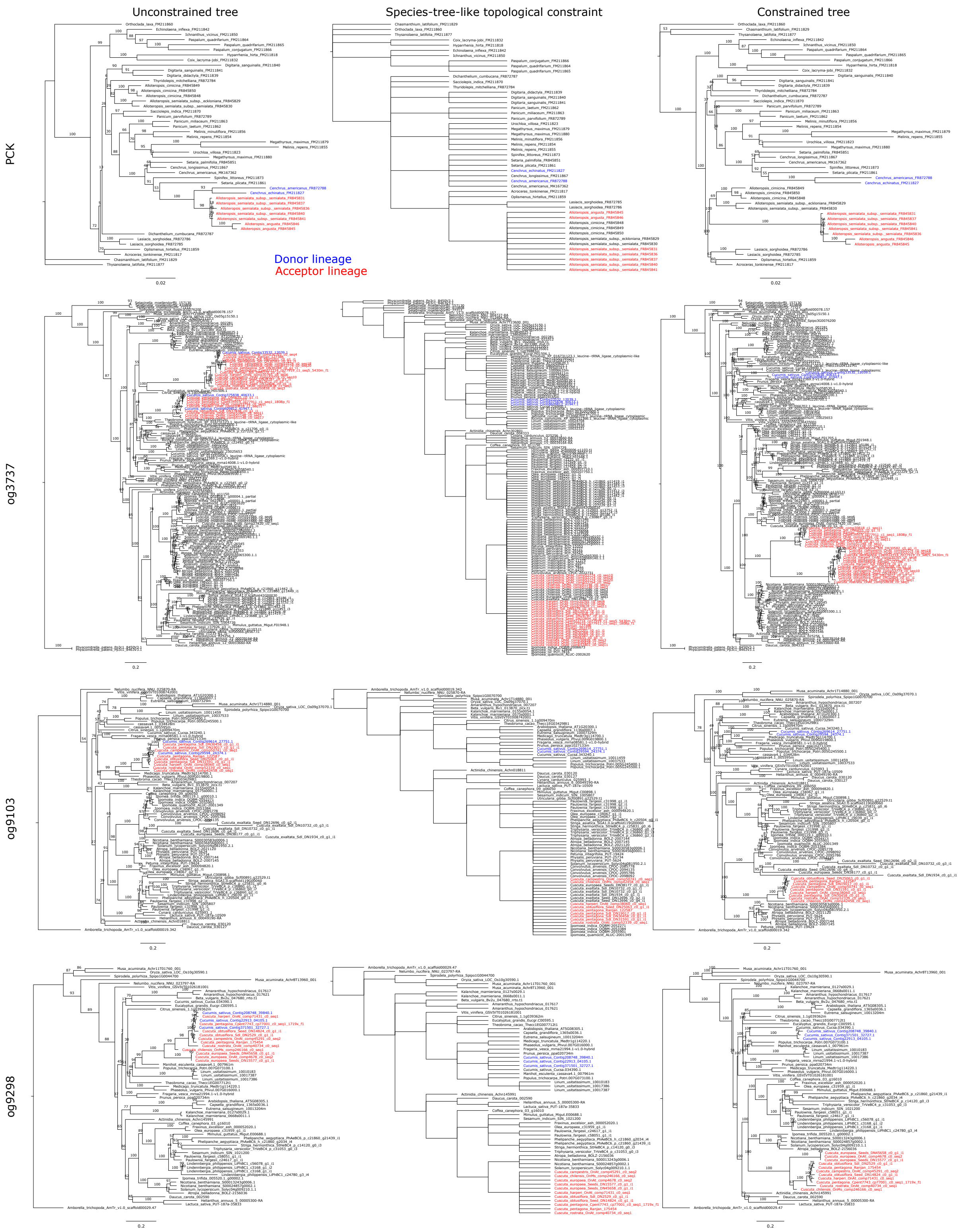

Supplementary Figure 7. Introducing the species-tree-like topology in the phylogenetic trees involving HGTs. Without a tree constraint, donors and acceptors form a sister clade in the maximum-likelihood phylogenetic analysis (left). When the taxonomic rank information is employed as a constraint in the topology inference (middle), the resulting trees inherit such topologies where donors and acceptors are separated (right). The constrained trees are used to examine how different metrics behave upon false convergence caused by the species-tree-like topology (Fig. 1e). Scale bars indicate substitutions per nucleotide site. Numbers on branches denote ultrafast bootstrapping values (also available as Newick files in Supplementary Dataset[94]).

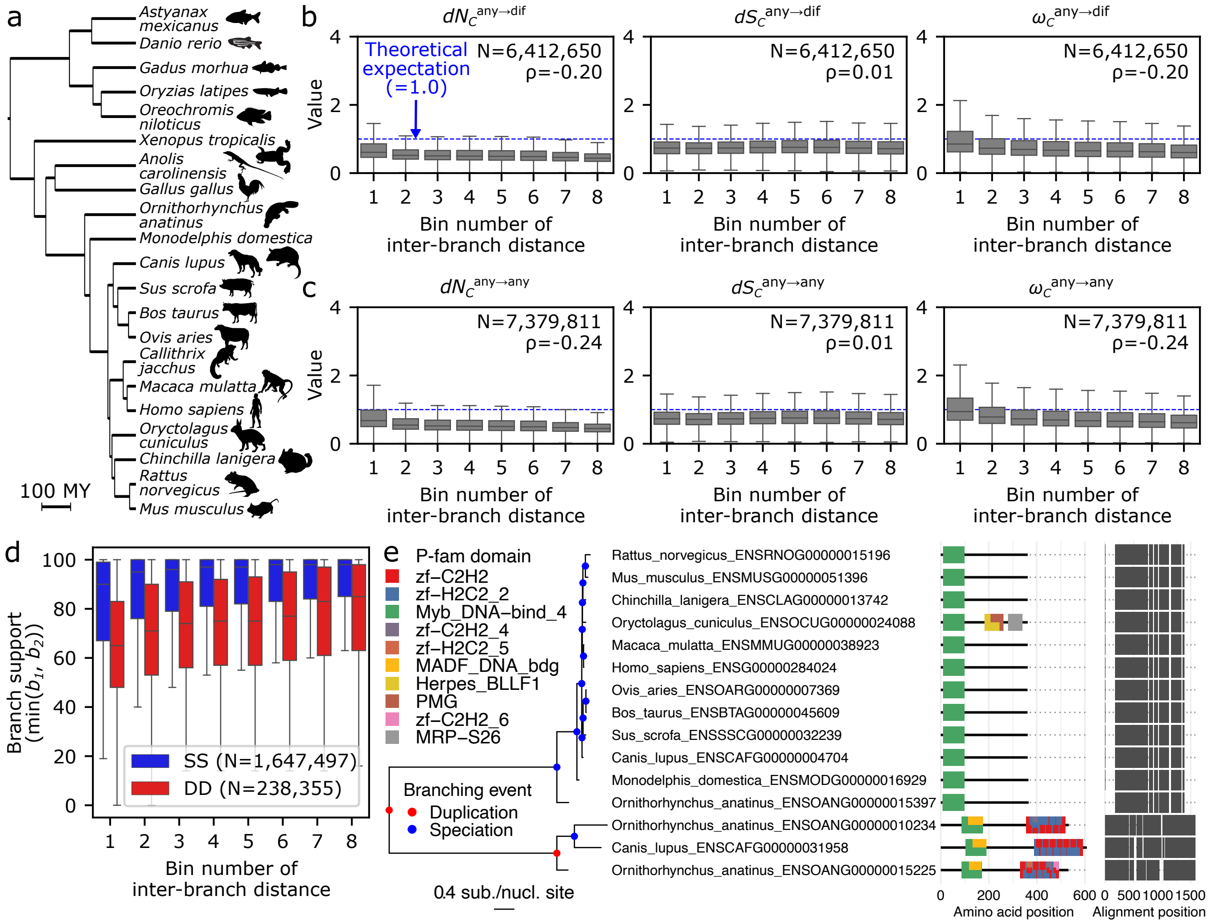

Supplementary Figure 8. Genome-scale analysis of convergence in nuclear-encoded genes. (**a**) The vertebrate species tree for the 21 analyzed genomes. Some animal silhouettes were obtained from PhyloPic (http://phylopic.org). The silhouettes of *Astyanax mexicanus* and *Oreochromis niloticus* are licensed under CC BY-NC-SA 3.0 (https://creativecommons.org/licenses/by-nc-sa/3.0/) by Milton Tan (reproduced with permission), that of *Rattus norvegicus* are licensed under CC BY-SA 3.0 (https://creativecommons.org/licenses/by-sa/3.0/) by Rebecca Groom (modified with permission), and those of *Anolis carolinensis* and *Ornithorhynchus anatinus* are licensed under CC BY 3.0 (https://creativecommons.org/licenses/by/3.0/) by Sarah Werning. (**b**) Temporal variation of double divergence rates. The number of branch pairs (N) and Spearman's correlation coefficients (ρ) are provided in the plot. Boxplot elements are defined as follows: center line, median; box limits, upper and lower quartiles; whiskers, 1.5 × interquartile range. (**c**) Temporal variation of paired substitution rates. (**d**) Branch supports in relation to gene duplication. The IQ-TREE's ultrafast bootstrap values are compared. Reconciled branches were treated as no support (= 0). (**e**) An orthogroup that contains extremely large genetic distances. The gene tree of OG0007724 is shown as an example. Node colors in the trees indicate inferred branching events of speciation (blue) and gene duplication (red). Two clades are connected by an extremely long branch and have non-homologous sets of protein domains. The placement and identity of P-fam protein domains (E value < 0.01) are shown to the right of the tree.

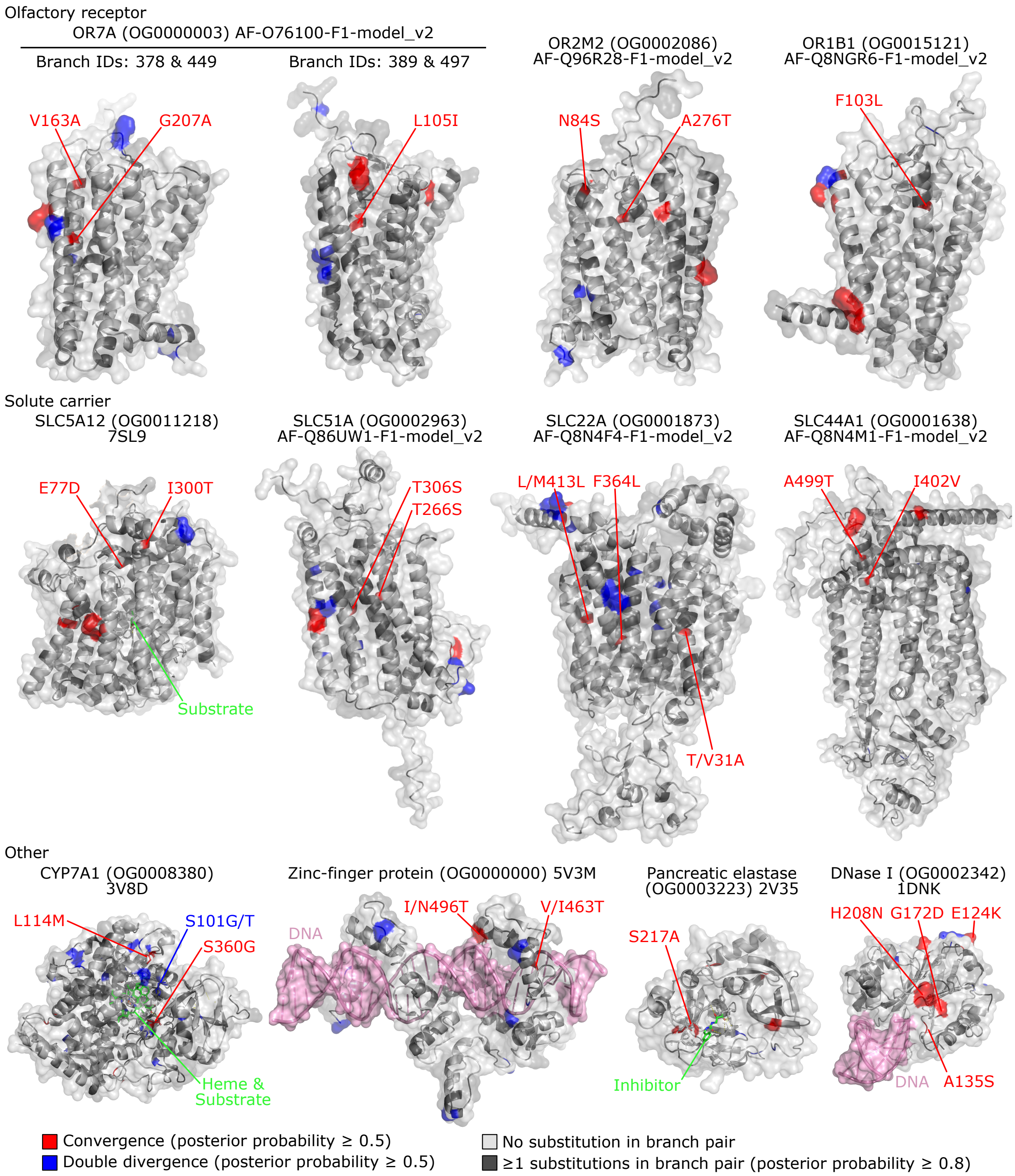

Supplementary Figure 9. Examples of proteins convergently evolved in herbivores. Convergently evolved proteins ($O_C^N \geq 3.0$and $\omega_C \geq 3.0$) in ruminants (*Bos taurus* and *Ovis aries*) and rabbits (*Oryctolagus cuniculus*) are shown (for a complete list, see Supplementary Table 5). Convergent amino acid substitutions discussed in the main text are labeled. Site numbers correspond to those in the PDB entry or the AlphaFold structure (accession numbers are indicated in the plot). Olfactory receptors and solute carriers are transmembrane proteins, and the upper portion of each protein corresponds to the extracellular region. The surface representation of the protein is overlaid with a cartoon representation. Convergent and divergent amino acid loci are highlighted in red and blue, respectively. Substrates and their analogs are shown as green sticks. Side chains forming the substrate-binding site are also shown as sticks. Note that these are the side chains in the protein from databases, so amino acid substitutions in the convergent lineages may result in distinct structures and arrangements.

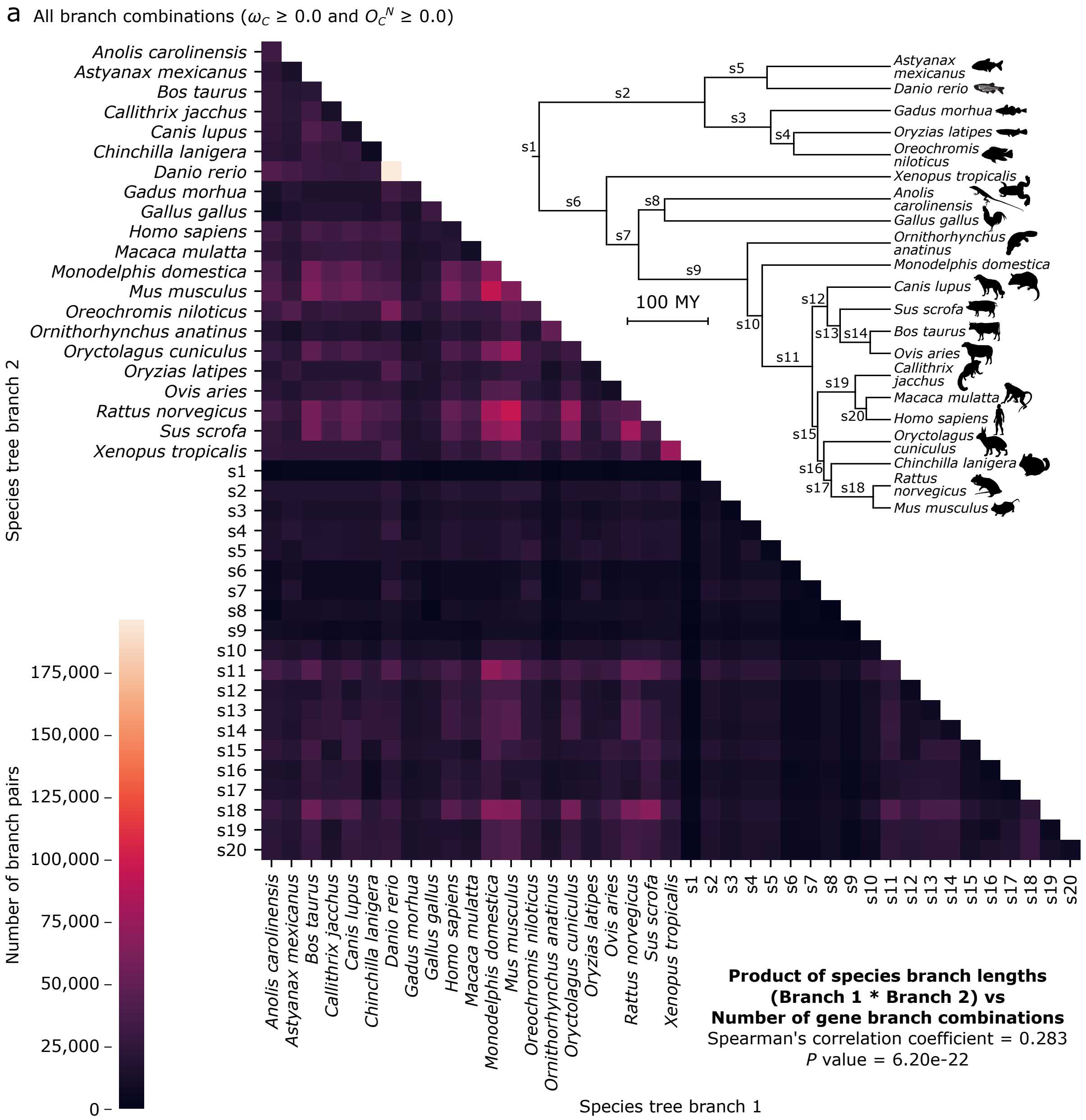

Supplementary Figure 10. Number of branch combinations in the animal genome analysis. (**a**) Number of gene branch pairs per species branch pair. Internal branch names are indicated in the species tree. No convergence threshold is applied (i.e., $\omega_C \geq 0.0$ and $O_C^N \geq 0.0$). Note that even identical branches or branches in an ancestor-descendant or sister relationship in the species tree can be independent in gene trees if there is a preceding gene duplication event. Some animal silhouettes were obtained from PhyloPic (http://phylopic.org). The silhouettes of *Astyanax mexicanus* and *Oreochromis niloticus* are licensed under CC BY-NC-SA 3.0 (https://creativecommons.org/licenses/by-nc-sa/3.0/) by Milton Tan (reproduced with permission), that of *Rattus norvegicus* are licensed under CC BY-SA 3.0 (https://creativecommons.org/licenses/by-sa/3.0/) by Rebecca Groom (modified with permission), and those of *Anolis carolinensis* and *Ornithorhynchus anatinus* are licensed under CC BY 3.0 (https://creativecommons.org/licenses/by/3.0/) by Sarah Werning. (**b**) Number of convergent gene branch pairs per species branch pair under the threshold of $\omega_C \geq 3.0$ and $O_C^N \geq 3.0$. (**c**) Relationships between the numbers of all branch pairs and convergent branch pairs. (**d**) Number of analyzed genes per genome.

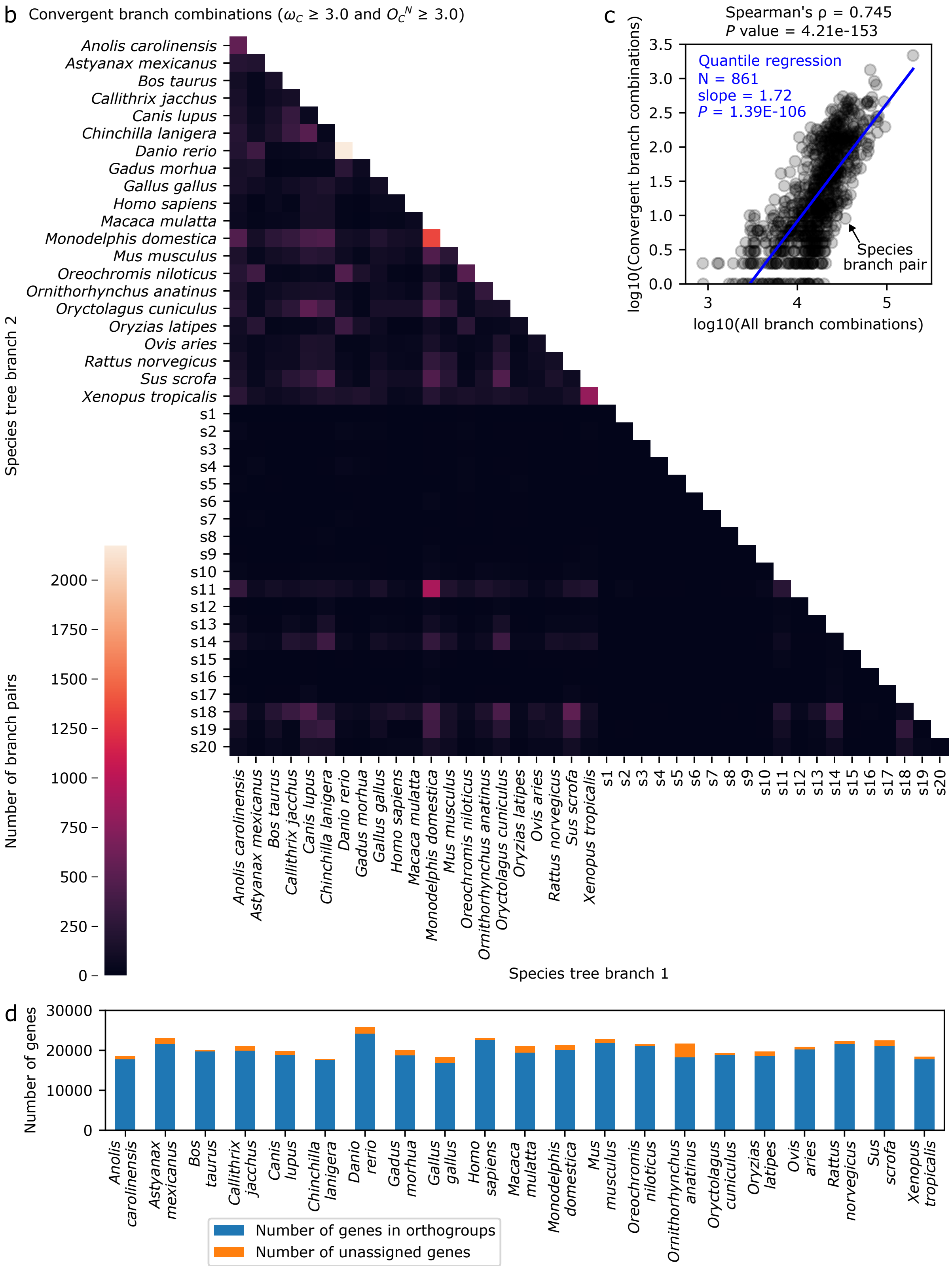

Supplementary Fig. 10 (continued)

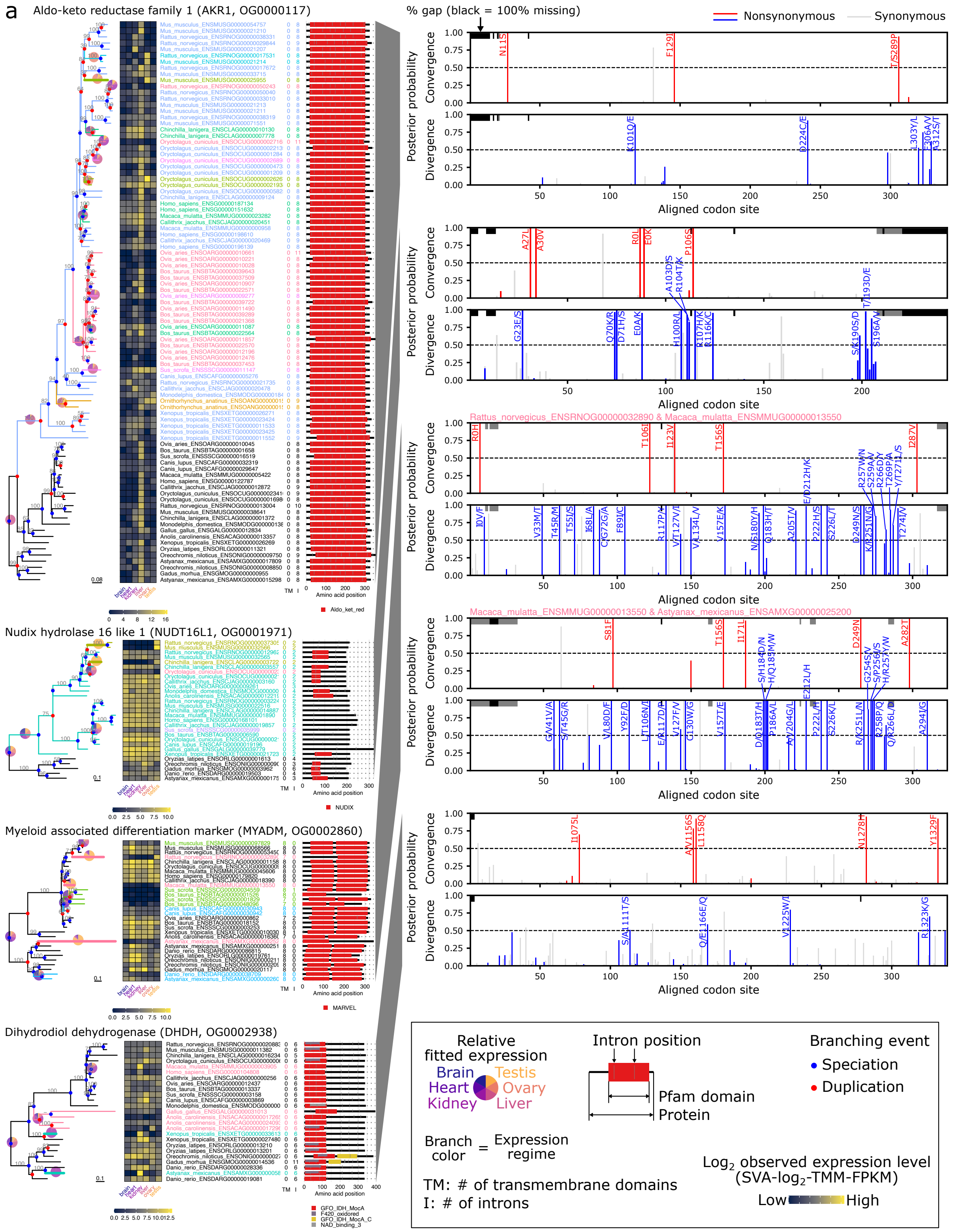

Supplementary Figure 11. Further characterization of protein convergence jointly occurring with gene expression convergence. (**a**) Complete phylogenetic trees and site-wise posterior probabilities of convergence and divergence in the detected branch pairs. IQ-TREE's ultrafast bootstrap values are shown above branches. A hyphen (-) marks a branch reconciled by GeneRax. Node colors in the trees indicate inferred branching events of speciation (blue) and gene duplication (red). The heatmap shows expression levels observed in extant species. The colors of branches and tip labels indicate expression regimes. Among-organ expression patterns are shown as a pie chart for each regime. Branches involved in joint convergence are highlighted with thick lines. To the right of the tip labels, the number of transmembrane domains

predicted by TMHMM[105], the number of introns in protein-coding sequences, and the Pfam domain structures (E-value < 0.01) are shown. Trees are available as pdf files in Supplementary Dataset[94]. (**b**) Hydrophobicity change of combinatorial amino acid substitutions. Theoretically derived hydrophobicity scales[151] were compared between the average values of ancestral and derived amino acids (Δ theoretical hydrophobicity; mean derived amino acid hydrophobicity – mean ancestral amino acid hydrophobicity). Convergent substitutions at the substrate-binding sites of DHDH are labeled and discussed in the main text. Boxplot elements are defined as follows: center line, median; box limits, upper and lower quartiles.

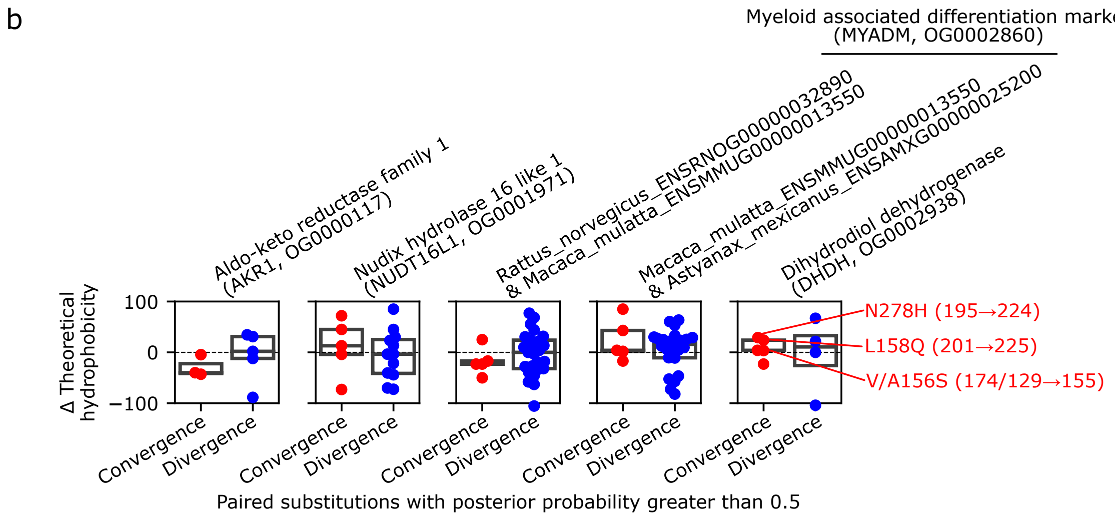

Supplementary Fig. 11 (continued)

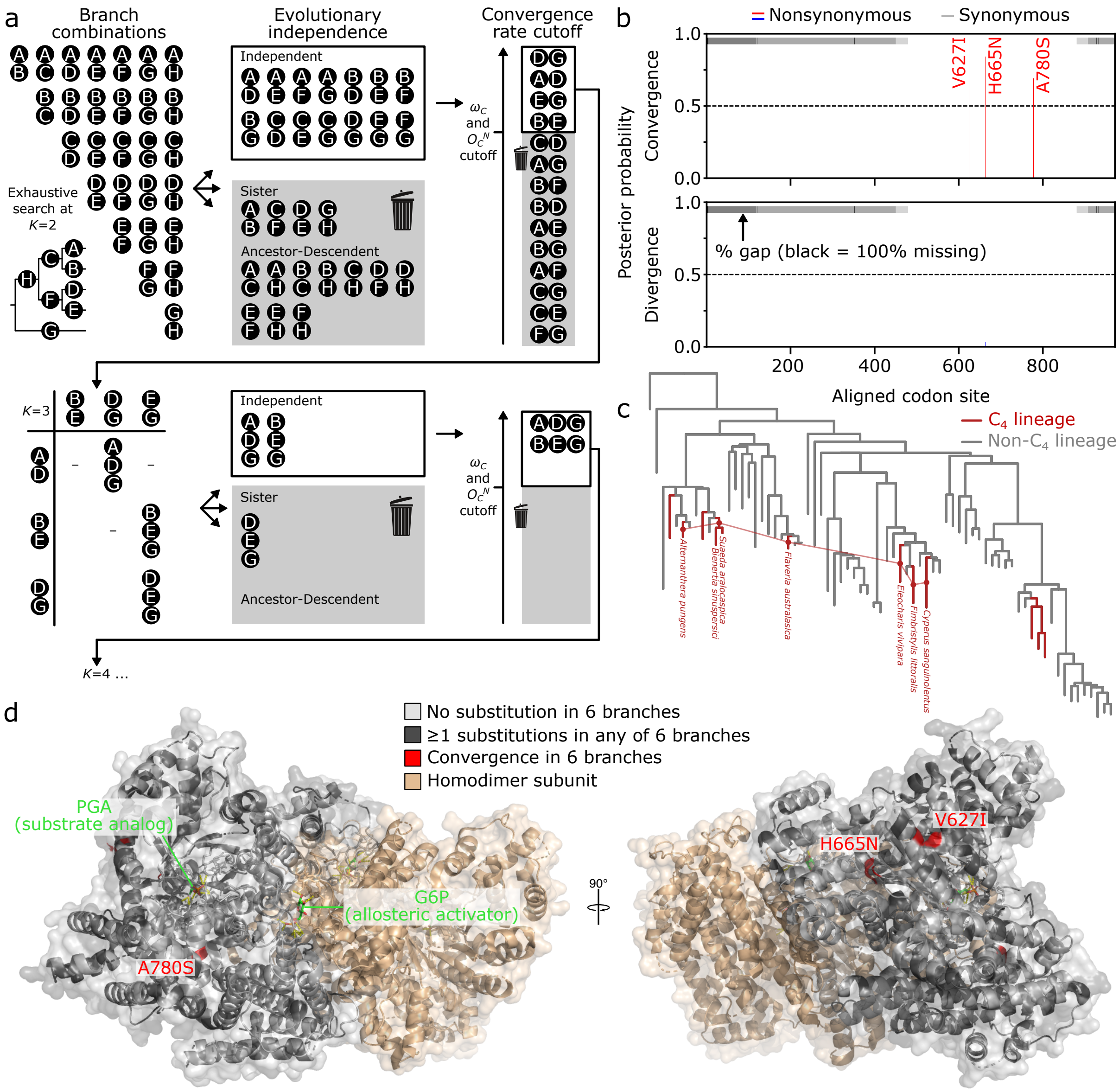

Supplementary Figure 12. Analysis of highly repetitive convergence. (**a**) Overview of the new branch-and-bound algorithm. This is a detailed illustration of Fig. 4a. (**b**) Site-specific probabilities of combinatorial substitutions in PEPC at $K = 6$. (**c**) Convergent branch combination in the PEPC tree at $K = 6$. (**d**) Positions of higher-order convergent substitutions in the structure of maize PEPC (PDB ID: 6MGI)[152]. Abbreviations: PGA, phosphoglycolate (substrate analog); G6P, glucose-6-phosphate (allosteric activator).

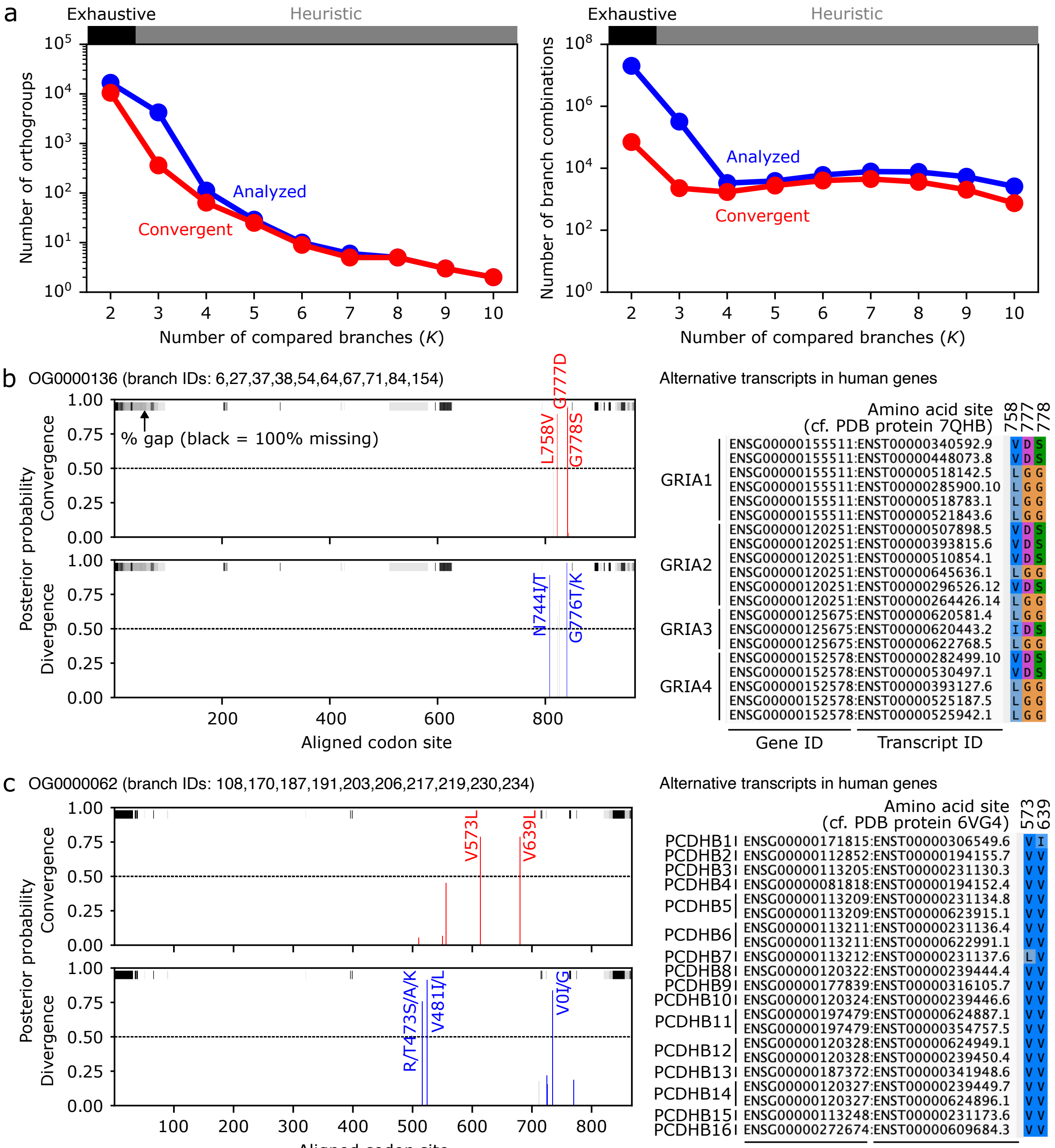

Supplementary Figure 13. Analysis of highly repetitive convergence in 21 animal genomes. (**a**) Numbers of orthogroups and branch combinations in the higher-order analysis. (**b**) Falsely detected protein convergence in OG0000136 at $K = 10$. Combinatorial substitutions are clustered to a limited range of the protein sequence. To the right, all alternative transcripts in human genes annotated in Ensembl are shown. Alternative transcripts from multiple genes harbor the same set of variations, likely generating false convergence. (**c**) Protein convergence in OG00000062 at $K = 10$. No evidence was found for shared variations among alternative transcripts. (**d**) Convergent branch combinations in the OG00000062 tree at $K = 6$. (**e**) Positions of higher-order convergent substitutions in the structure of a protocadherin ectodomain (PDB ID: 6VG4)[153].

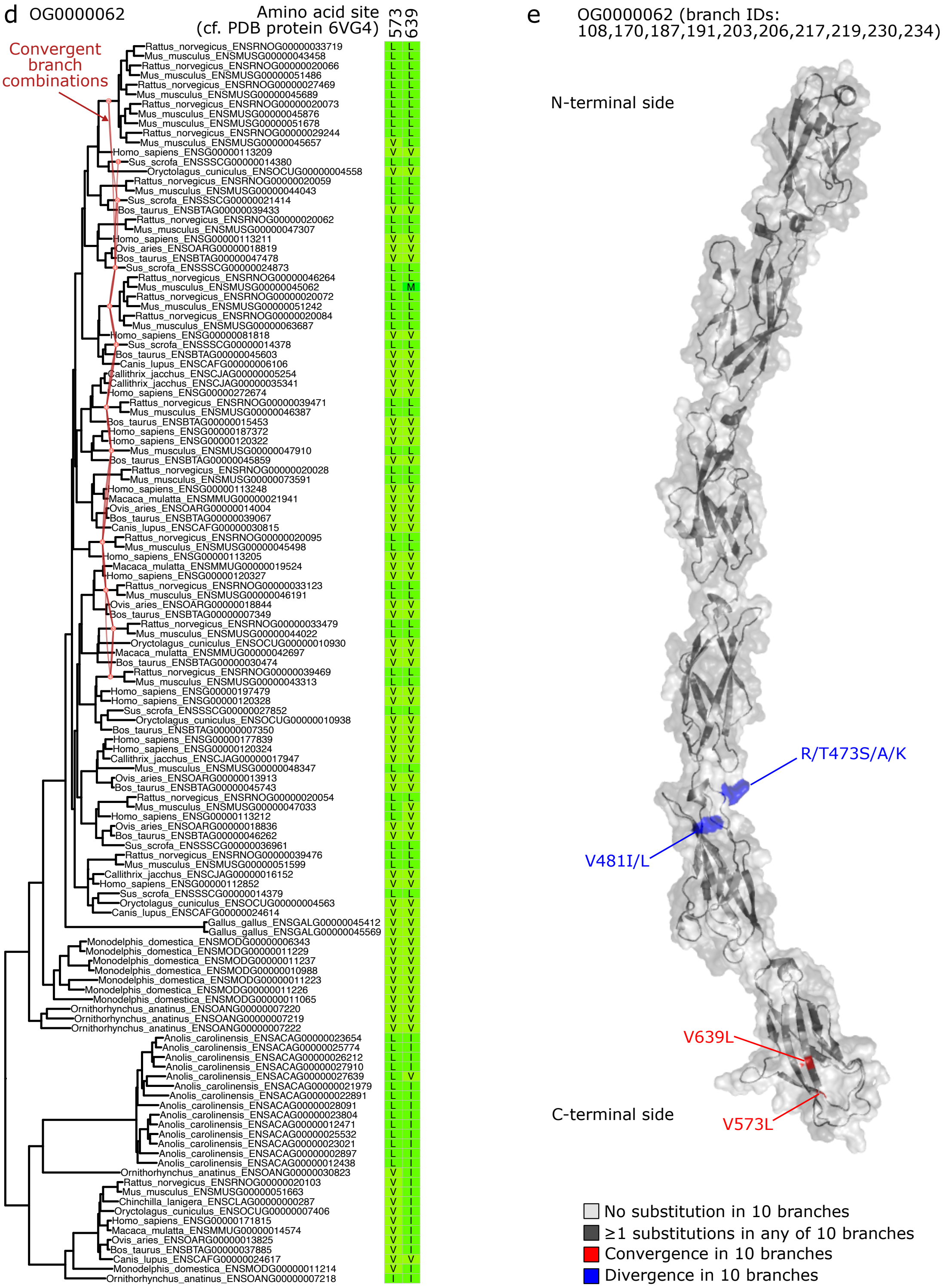

Supplementary Fig. 13 (continued)

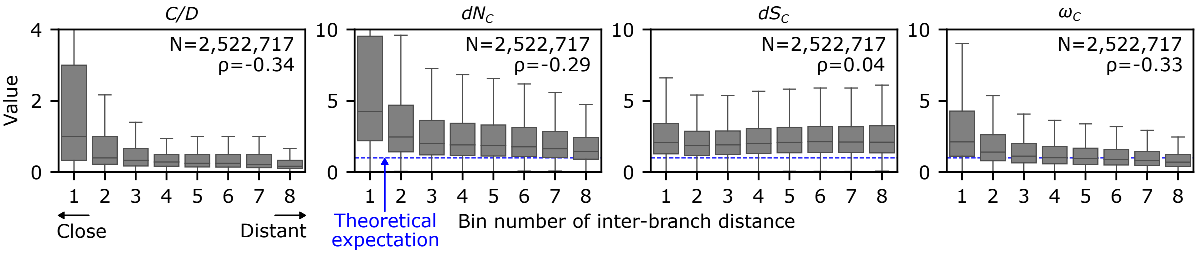

Supplementary Figure 14. Temporal variation of convergence rates, as estimated with the binarized probabilities of ancestral states. The analysis of Fig. 2b is reproduced with the --ml_anc option in CSUBST. The number of branch pairs (N) and Spearman's correlation coefficients (ρ) are provided in each plot. The bin range was determined to assign an equal number of branch pairs. To reduce the noise originating from branches where almost no substitutions occurred, branch pairs with both O_C^N and O_C^S greater than or equal to 1.0 were analyzed. Boxplot elements are defined as follows: center line, median; box limits, upper and lower quartiles; whiskers, 1.5 × interquartile range.

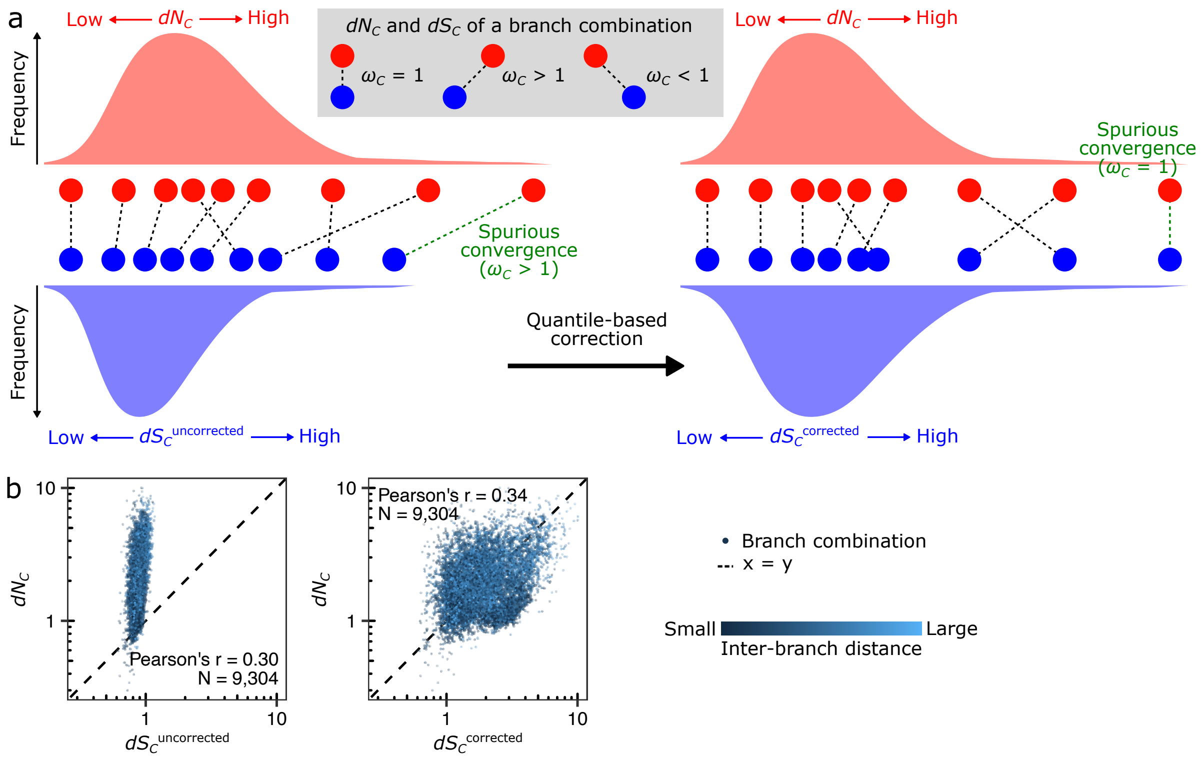

Supplementary Figure 15. The long-tail correction matches the range of distributions between dN_C and dS_C. (**a**) A schematic representation of the long-tail correction (Equation 18). (**b**) Calibration of synonymous convergence rates in mitochondrial proteins. The mitochondrial genome data in Fig. 1e was analyzed. The inter-branch distance is shown on a color scale. The number of branch pairs (N) and Pearson's correlation coefficients (r) are provided in the plot.

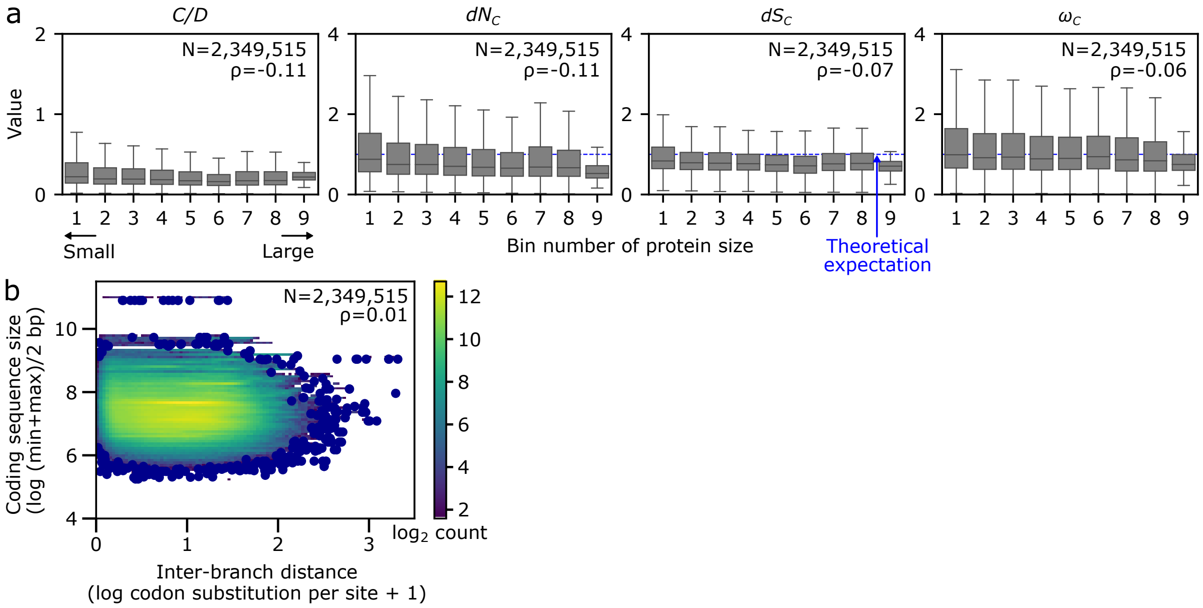

Supplementary Figure 16. Relationships between protein sizes and convergence rates in vertebrate nucleus-encoded genes. (**a**) Protein-size-dependent variation of convergence rates. The bin range was determined to assign an equal number of branch pairs. Boxplot elements are defined as follows: center line, median; box limits, upper and lower quartiles; whiskers, 1.5 × interquartile range. (**b**) Relationships between genetic distance and the size of proteins. While the inter-branch distance was obtained for each branch pair, the coding sequence size was defined for each orthogroup.

Supplementary References

98. Yates, A. *et al.* Ensembl 2016. *Nucleic Acids Res.* **44**, D710–D716 (2016).
99. Simão, F. A., Waterhouse, R. M., Ioannidis, P., Kriventseva, E. V. & Zdobnov, E. M. BUSCO: assessing genome assembly and annotation completeness with single-copy orthologs. *Bioinformatics* **31**, 3210–3212 (2015).
100. Emms, D. M. & Kelly, S. OrthoFinder: solving fundamental biases in whole genome comparisons dramatically improves orthogroup inference accuracy. *Genome Biol.* **16**, 157 (2015).
101. Grabherr, M. G. *et al.* Full-length transcriptome assembly from RNA-Seq data without a reference genome. *Nat. Biotechnol.* **29**, 644–652 (2011).
102. Chen, S., Zhou, Y., Chen, Y. & Gu, J. fastp: an ultra-fast all-in-one FASTQ preprocessor. *Bioinformatics* **34**, i884–i890 (2018).
103. Camacho, C. *et al.* BLAST+: architecture and applications. *BMC Bioinformatics* **10**, 421 (2009).
104. El-Gebali, S. *et al.* The Pfam protein families database in 2019. *Nucleic Acids Res.* **47**, D427–D432 (2019).
105. Krogh, A., Larsson, B., von Heijne, G. & Sonnhammer, E. L. L. Predicting transmembrane protein topology with a hidden Markov model: application to complete genomes. *J. Mol. Biol.* **305**, 567–580 (2001).
106. Rice, P., Longden, I. & Bleasby, A. EMBOSS: the European Molecular Biology Open Software Suite. *Trends Genet.* **16**, 276–277 (2000).
107. Steenwyk, J. L., Iii, T. J. B., Li, Y., Shen, X.-X. & Rokas, A. ClipKIT: A multiple sequence alignment trimming software for accurate phylogenomic inference. *PLOS Biol.* **18**, e3001007 (2020).
108. Nguyen, L.-T., Schmidt, H. A., von Haeseler, A. & Minh, B. Q. IQ-TREE: A fast and effective stochastic algorithm for estimating maximum-likelihood phylogenies. *Mol. Biol. Evol.* **32**, 268–274 (2015).
109. Yang, Z. PAML 4: phylogenetic analysis by maximum likelihood. *Mol. Biol. Evol.* **24**, 1586–1591 (2007).
110. Zhang, G.-Q. *et al.* The *Apostasia* genome and the evolution of orchids. *Nature* **549**, 379–383 (2017).
111. Yu, G., Smith, D. K., Zhu, H., Guan, Y. & Lam, T. T.-Y. GGTREE: an R package for visualization and annotation of phylogenetic trees with their covariates and other associated data. *Methods Ecol. Evol.* **8**, 28–36 (2017).
112. Hunter, J. D. Matplotlib: a 2D graphics environment. *Comput. Sci. Eng.* **9**, 90–95 (2007).
113. Waskom, M. L. seaborn: statistical data visualization. *J. Open Source Softw.* **6**, 3021 (2021).
114. Wickham, H. *ggplot2: Elegant Graphics for Data Analysis*. (Springer-Verlag, 2009). doi:10.1007/978-0-387-98141-3.
115. Parker, J. *et al.* Genome-wide signatures of convergent evolution in echolocating mammals. *Nature* **502**, 228–231 (2013).
116. Pollock, D. D. & Pollard, S. T. Parallel and convergent molecular evolution. in *Encyclopedia of Evolutionary Biology* (ed. Kliman, R. M.) 206–211 (Academic Press, 2016).
117. Goldstein, R. A. & Pollock, D. D. Sequence entropy of folding and the absolute rate of amino acid substitutions. *Nat. Ecol. Evol.* **1**, 1923–1930 (2017).
118. Arendt, J. & Reznick, D. Convergence and parallelism reconsidered: what have we learned about the genetics of adaptation? *Trends Ecol. Evol.* **23**, 26–32 (2008).
119. Pond, S. L. K. & Frost, S. D. W. Not so different after all: A comparison of methods for detecting amino acid sites under selection. *Mol. Biol. Evol.* **22**, 1208–1222 (2005).
120. Burskaia, V. *et al.* Excessive parallelism in protein evolution of Lake Baikal amphipod species

flock. *Genome Biol. Evol.* **12**, 1493–1503 (2020).
121. Jones, D. T., Taylor, W. R. & Thornton, J. M. The rapid generation of mutation data matrices from protein sequences. *Comput. Appl. Biosci.* **8**, 275–282 (1992).
122. Lartillot, N. & Philippe, H. A Bayesian mixture model for across-site heterogeneities in the amino-acid replacement process. *Mol. Biol. Evol.* **21**, 1095–1109 (2004).
123. Zou, Z. & Zhang, J. Gene tree discordance does not explain away the temporal decline of convergence in mammalian protein sequence evolution. *Mol. Biol. Evol.* **34**, 1682–1688 (2017).
124. Pollock, D. D., Thiltgen, G. & Goldstein, R. A. Amino acid coevolution induces an evolutionary Stokes shift. *Proc. Natl. Acad. Sci. U. S. A.* **109**, E1352–E1359 (2012).
125. Shah, P., McCandlish, D. M. & Plotkin, J. B. Contingency and entrenchment in protein evolution under purifying selection. *Proc. Natl. Acad. Sci. U. S. A.* **112**, E3226–E3235 (2015).
126. Starr, T. N., Flynn, J. M., Mishra, P., Bolon, D. N. A. & Thornton, J. W. Pervasive contingency and entrenchment in a billion years of Hsp90 evolution. *Proc. Natl. Acad. Sci. U. S. A.* **115**, 4453–4458 (2018).
127. Conant, G. C. & Wolfe, K. H. Turning a hobby into a job: How duplicated genes find new functions. *Nat. Rev. Genet.* **9**, 938–950 (2008).
128. Rižner, T. L. & Penning, T. M. Role of aldo–keto reductase family 1 (AKR1) enzymes in human steroid metabolism. *Steroids* **79**, 49–63 (2014).
129. Couture, J.-F. *et al.* Loop relaxation, a mechanism that explains the reduced specificity of rabbit 20α-hydroxysteroid dehydrogenase, a member of the aldo-keto reductase superfamily. *J. Mol. Biol.* **339**, 89–102 (2004).
130. Gunaratne, J. *et al.* Protein interactions of phosphatase and tensin homologue (PTEN) and its cancer-associated G20E mutant compared by using stable isotope labeling by amino acids in cell culture-based parallel affinity purification. *J. Biol. Chem.* **286**, 18093–18103 (2011).
131. Botuyan, M. V. *et al.* Mechanism of 53BP1 activity regulation by RNA-binding TIRR and a designer protein. *Nat. Struct. Mol. Biol.* **25**, 591–600 (2018).
132. Thirawatananond, P. *et al.* Structural analyses of NudT16–ADP-ribose complexes direct rational design of mutants with improved processing of poly(ADP-ribosyl)ated proteins. *Sci. Rep.* **9**, 5940 (2019).
133. Zhang, F. *et al.* Nudix hydrolase NUDT16 regulates 53BP1 protein by reversing 53BP1 ADP-ribosylation. *Cancer Res.* **80**, 999–1010 (2020).
134. Aranda, J. F. *et al.* MYADM regulates Rac1 targeting to ordered membranes required for cell spreading and migration. *Mol. Biol. Cell* **22**, 1252–1262 (2011).
135. Dy, A. B. C. *et al.* Myeloid-associated differentiation marker is a novel SP-A-associated transmembrane protein whose expression on airway epithelial cells correlates with asthma severity. *Sci. Rep.* **11**, 23392 (2021).
136. Sun, L. *et al.* Oncological miR-182-3p, a novel smooth muscle cell phenotype modulator, evidences from model rats and patients. *Arterioscler. Thromb. Vasc. Biol.* **36**, 1386–1397 (2016).
137. Terada, A., Okada-Hatakeyama, M., Tsuda, K. & Sese, J. Statistical significance of combinatorial regulations. *Proc. Natl. Acad. Sci. U. S. A.* **110**, 12996–13001 (2013).
138. Marcovitz, A. *et al.* A functional enrichment test for molecular convergent evolution finds a clear protein-coding signal in echolocating bats and whales. *Proc. Natl. Acad. Sci. U. S. A.* **116**, 21094–21103 (2019).
139. Rey, C., Guéguen, L., Sémon, M. & Boussau, B. Accurate detection of convergent amino-acid evolution with PCOC. *Mol. Biol. Evol.* **35**, 2296–2306 (2018).
140. Kowalczyk, A. *et al.* RERconverge: an R package for associating evolutionary rates with convergent traits. *Bioinformatics* **35**, 4815–4817 (2019).
141. Hu, Z., Sackton, T. B., Edwards, S. V. & Liu, J. S. Bayesian detection of convergent rate changes

of conserved noncoding elements on phylogenetic trees. *Mol. Biol. Evol.* **36**, 1086–1100 (2019).
142. Hiller, M. *et al.* A "Forward Genomics" approach links genotype to phenotype using independent phenotypic losses among related species. *Cell Rep.* **2**, 817–823 (2012).
143. Prudent, X. *et al.* Controlling for phylogenetic relatedness and evolutionary rates improves the discovery of associations between species' phenotypic and genomic differences. *Mol. Biol. Evol.* **33**, 2135–2150 (2016).
144. Marcovitz, A., Jia, R. & Bejerano, G. "Reverse Genomics" predicts function of human conserved noncoding elements. *Mol. Biol. Evol.* **33**, 1358–1369 (2016).
145. Anzalone, A. V., Koblan, L. W. & Liu, D. R. Genome editing with CRISPR–Cas nucleases, base editors, transposases and prime editors. *Nat. Biotechnol.* **38**, 824–844 (2020).
146. Knott, G. J. & Doudna, J. A. CRISPR-Cas guides the future of genetic engineering. *Science* **361**, 866–869 (2018).
147. Chandler, C. H., Chari, S. & Dworkin, I. Does your gene need a background check? How genetic background impacts the analysis of mutations, genes, and evolution. *Trends Genet.* **29**, 358–366 (2013).
148. Karageorgi, M. *et al.* Genome editing retraces the evolution of toxin resistance in the monarch butterfly. *Nature* **574**, 409–412 (2019).
149. Taverner, A. M. *et al.* Adaptive substitutions underlying cardiac glycoside insensitivity in insects exhibit epistasis in vivo. *eLife* **8**, e48224 (2019).
150. Lyons, D. M., Zou, Z., Xu, H. & Zhang, J. Idiosyncratic epistasis creates universals in mutational effects and evolutionary trajectories. *Nat. Ecol. Evol.* **4**, 1685–1693 (2020).
151. Tien, M. Z., Meyer, A. G., Sydykova, D. K., Spielman, S. J. & Wilke, C. O. Maximum allowed solvent accessibilites of residues in proteins. *PloS One* **8**, e80635 (2013).
152. Muñoz-Clares, R. A., González-Segura, L., Juárez-Díaz, J. A. & Mújica-Jiménez, C. Structural and biochemical evidence of the glucose 6-phosphate-allosteric site of maize C_4-phosphoenolpyruvate carboxylase: its importance in the overall enzyme kinetics. *Biochem. J.* **477**, 2095–2114 (2020).
153. Harrison, O. J. *et al.* Family-wide structural and biophysical analysis of binding interactions among non-clustered δ-protocadherins. *Cell Rep.* **30**, 2655-2671.e7 (2020).
